# The thylakoid- and pyrenoid-localized phosphate transporter PHT4-9 is essential for photosynthesis in Chlamydomonas

**DOI:** 10.1093/plphys/kiaf158

**Published:** 2025-04-24

**Authors:** Kashif Mohd Shaikh, Charlotte E Walker, Dávid Tóth, Soujanya Kuntam, Tamás F Polgár, Nia Z Petrova, Herbie Garland, Luke C M Mackinder, Szilvia Z Tóth, Cornelia Spetea

**Affiliations:** Department of Biological and Environmental Sciences, University of Gothenburg, Gothenburg 40530, Sweden; Institute of Plant Biology, HUN-REN Biological Research Centre, Szeged, Szeged H-6726, Hungary; Centre for Novel Agricultural Products (CNAP), Department of Biology, University of York, Heslington, York YO10 5DD, UK; Institute of Plant Biology, HUN-REN Biological Research Centre, Szeged, Szeged H-6726, Hungary; Institute of Plant Biology, HUN-REN Biological Research Centre, Szeged, Szeged H-6726, Hungary; Institute of Biophysics, HUN-REN Biological Research Centre, Szeged, Szeged H-6726, Hungary; Institute of Plant Biology, HUN-REN Biological Research Centre, Szeged, Szeged H-6726, Hungary; Centre for Novel Agricultural Products (CNAP), Department of Biology, University of York, Heslington, York YO10 5DD, UK; Centre for Novel Agricultural Products (CNAP), Department of Biology, University of York, Heslington, York YO10 5DD, UK; Institute of Plant Biology, HUN-REN Biological Research Centre, Szeged, Szeged H-6726, Hungary; Department of Biological and Environmental Sciences, University of Gothenburg, Gothenburg 40530, Sweden

## Abstract

Phosphate (Pi) is essential for photosynthesis in the chloroplast of algae and plants. Pi homeostasis in the chloroplast is maintained by transporters from several families, whose identities in algae are largely unknown as compared with land plants. Here, we assess the role of the putative PHOSPHATE TRANSPORTER 4-9 from *Chlamydomonas reinhardtii* (CrPHT4-9) in maintaining chloroplast Pi homeostasis and modulating photosynthesis. Based on phylogenetic analyses and heterologous expression in a yeast (*Saccharomyces cerevisiae*) strain lacking Pi transporters, we demonstrate that CrPHT4-9 is a Pi transporter closely related to the chloroplast members of the PHT4 family in Arabidopsis (*Arabidopsis thaliana*). CrPHT4-9 is localized within the chloroplast, more specifically in the thylakoid membrane network and the tubules traversing the CO_2_-fixing pyrenoid. Two mutants lacking CrPHT4-9 (*Crpht4-9*) exhibit defective photoautotrophic growth, altered cell morphology and chloroplast ultrastructure under CO_2_-limiting conditions. In the *Crpht4-9* mutants, we further show an increased proton motive force across the thylakoid membrane, enhanced energy- and state-transition-dependent non-photochemical quenching of chlorophyll *a* fluorescence, and diminished photosynthetic electron transport and ATP synthase activity. The *Crpht4-9* mutants exhibit reduced affinity to inorganic carbon, indicating an impaired carbon-concentrating mechanism. These phenotypes are largely recovered by genetic complementation as well as by ample CO_2_ supply and, interestingly, by Pi deprivation. Therefore, we conclude that the thylakoid- and pyrenoid-localized CrPHT4-9 maintains Pi homeostasis within the chloroplast and is essential for photosynthesis and growth.

## Teaser

In Chlamydomonas, the thylakoid- and pyrenoid-localized phosphate transporter PHT4-9 is essential for maintaining chloroplast phosphate homeostasis, thus supporting efficient photosynthesis and growth.

## Introduction

Photosynthesis is a key biological process to life on Earth as it provides energy, oxygen, and food for most living organisms. In land plants and eukaryotic algae, this process takes place inside the chloroplast, and it involves extensive ion and metabolite transport across the envelope and thylakoid membranes. Little is known about chloroplast channels and transporters playing this important role in algae as compared with land plants (Marchand et al. 2018). Even though many of the plant homologs have been predicted in various algal genomes, the few characterized chloroplast transporters are mostly from *Chlamydomonas reinhardtii* (hereafter Chlamydomonas).

In the chloroplast, phosphate (Pi) is required to synthesize ATP during photosynthesis, which in turn drives carbon fixation and regulates the activity of various proteins by phosphorylation. Pi is also a component of nucleic acids produced by the chloroplast genome. It is well known that Pi deficiency in green algae and land plants adversely affects growth, cell composition, and various aspects of photosynthesis, such as the synthesis of photosynthetic pigments, the efficiency of light harvesting, the rate of photosynthetic electron transport, non-photochemical quenching, the maximum rates of Rubisco carboxylation and ribulose-1,5-bisphosphate regeneration (Huesemann et al. 2013; Yan et al. 2015; Ota et al. 2016; Carstensen et al. 2018; Yang et al. 2018; Matsui et al. 2020; Kumari et al. 2021). Therefore, it is crucial to maintain an optimal Pi concentration in the chloroplast to ensure an efficient photosynthetic performance.

In the chloroplast of land plants, Pi homeostasis is maintained by plastidic PHOSPHATE TRANSLOCATORS (pPT) and by members of the PHOSPHATE TRANSPORTER 2 and 4 (PHT2 and PHT4) families (Weber et al. 2005; Versaw and Garcia 2017), but their counterparts in algae are largely unknown (Weber et al. 2005; Marchand et al. 2018; Wang et al. 2020). pPTs import cytosolic Pi into the chloroplast stroma in exchange for several types of phosphorylated photo-assimilates, namely triose-Pi, phosphoenolpyruvate, glucose 6-Pi, and xylulose-5-Pi translocators. All pPT types were found in genomes of green algae (Weber and Linka 2011). One triose-Pi/Pi translocator was found in the chloroplast envelope of Chlamydomonas and was proposed to export excess photo-assimilates out of the chloroplast and to prevent oxidative stress (Huang et al. 2023).

PHT2 and PHT4 proteins from *Arabidopsis thaliana* (hereafter Arabidopsis) transport Pi in symport with cations (H^+^, Na^+^), as evidenced from yeast and bacterial heterologous expression (Versaw and Harrison 2002; Guo et al. 2008; Pavon et al. 2008). AtPHT2;1 was localized to the envelope and found to be important for Pi allocation in Pi starvation conditions in plants (Versaw and Harrison 2002). Sequence homologs of AtPHT2;1 were found in carophytes but not in unicellular algae such as Chlamydomonas (Bonnot et al. 2017).

Among PHT4s in Arabidopsis, AtPHT4;1 is located in the chloroplast thylakoid membrane (Pavon et al. 2008) where it locally supplies Pi to the ATP synthase during the photochemical reactions (Karlsson et al. 2015), whereas AtPHT4;4 resides in the chloroplast envelope and transports ascorbate into the chloroplast stroma (Guo et al. 2008; Miyaji et al. 2015; Nam et al. 2021; Raju et al. 2024; Tóth et al. 2024b). AtPHT4;3 and AtPHT4;5 are also located in the envelope (Guo et al. 2008). AtPHT4;3 was found important to be a key player in the reduction of stromal Pi levels at high CO_2_ (Bouain et al. 2022), whereas AtPHT4;5 was found to enhance stromal Pi levels in drought and oxidative stress (Liu et al. 2023). AtPHT4;6 is a Golgi-located Pi transporter involved in salt tolerance and disease resistance (Hassler et al. 2012). Phylogenetic analyses of PHT4s revealed many homolog sequences in the genomes of green algae (Pfeil et al. 2014; Wang et al. 2020). Recently, the CrPHT4-7 transporter was localized to the chloroplast envelope and found important for Pi homeostasis and photosynthesis (Tóth et al. 2024a). However, the mechanism of Pi transport across the thylakoid membrane and the pyrenoid-traversing tubules remains unclear, despite the presence of key proteins undergoing phosphorylation/dephosphorylation within the thylakoid lumen and pyrenoid matrix, which are essential for pyrenoid function in CO_2_ assimilation (for reviews, see He et al. (2023); Catherall et al. (2025)).

During our search for potential chloroplast transporters from Chlamydomonas involved in Pi fluxes across the thylakoid membrane, we identified CrPHT4-9, another member of the PHT4 family. This identification was based on the growth defects of 2 Chlamydomonas Library Project (CLiP) *pht4-9* mutants in CO_2_-concentrating mechanism (CCM) and photosynthesis pooled library screens (Li et al. 2019; Fauser et al. 2022).

In this work, we demonstrate that CrPHT4-9 improves the growth of a yeast mutant lacking 5 endogenous Pi transporters. We further show that the protein is localized within the chloroplast, more specifically in the thylakoid membrane network and the tubules traversing the CO_2_-fixing pyrenoid. Two mutants lacking CrPHT4-9 (*pht4-9*) exhibit defective photoautotrophic growth, altered cell morphology and chloroplast ultrastructure under CO_2_-limiting conditions. In the *pht4-9* mutants, we further found increased proton motive force (PMF) across the thylakoid membrane, enhanced energy- and state-transition-dependent non-photochemical chlorophyll *a* fluorescence quenching (NPQ), and diminished photosynthetic electron transport and ATP synthase activity. The *pht4-9* mutants exhibited elevated half-saturation constants for inorganic carbon (*C_i_*), suggesting impaired CCM. These phenotypes were largely recovered by Pi deprivation and high CO_2_ supply. Finally, we compare the observed phenotype of the *pht4-9* mutants with those previously reported for the Arabidopsis mutants lacking the thylakoid Pi transporter AtPHT4;1 (Karlsson et al. 2015) and for Chlamydomonas mutants of the envelope Pi transporter CrPHT4-7 (Tóth et al. 2024a).

## Results

### Chlamydomonas PHT4-9 is closely related to the chloroplast members of the PHT4 family in Arabidopsis

**Figure 1.**
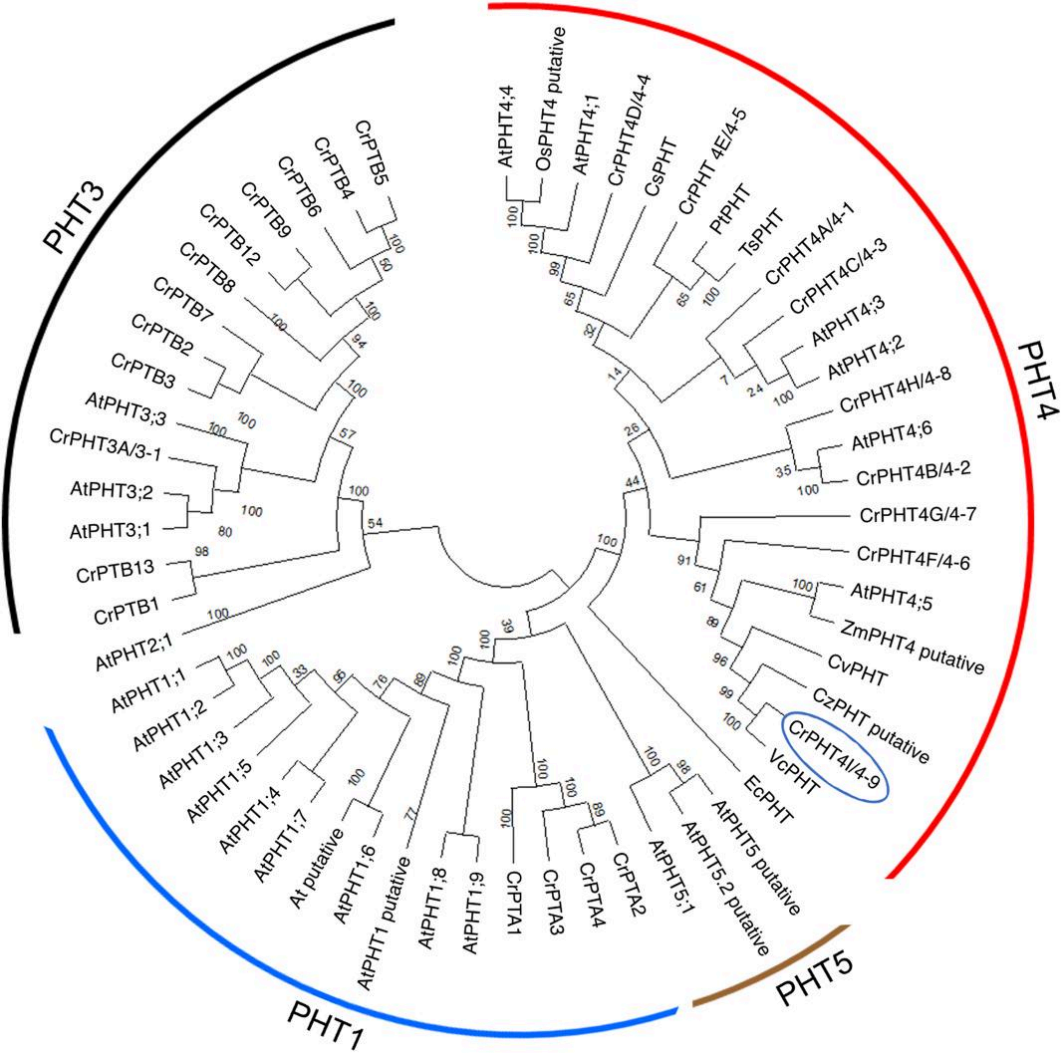
PHT4-9 from Chlamydomonas is a putative chloroplast Pi transporter conserved in the green lineage. Phylogenetic tree of the full-length CrPHT4-9/4I protein (name in circle) with known and putative PHTs from various land plants, green algae, diatoms and bacteria is shown. The evolutionary history was inferred using the Neighbor-Joining method with 500 bootstraps and the default settings of MEGA11 and the optimal tree is shown. The tree is divided into 4 groups: PHT1, PHT3, PHT4, and PHT5. The evolutionary distances are indicated by the branch lengths measured in the number of amino acid substitutions per site. At, *Arabidopsis thaliana*; Os, *Oryza sativa*; Zm, *Zea mays* (land plants); Cr, *Chlamydomonas reinhardtii*; Cz, *Chlorella zofingiensis*; Cv, *Chlorella vulgaris*; Vc, *Volvox carteri* (green algae); Cm, *Cyanidioschyzon merolae* (red alga); Pt, *Phaeodactylum tricornutum*; Ts, *Thalassiosira pseudonana* (diatoms); Ec, *Escherichia coli*.

There are 4 well-characterized families of Pi transporters (PHT1 to 4) in land plants, whereas in green algae they are poorly studied. Wang et al. (2020) found 25 putative PHTs in the Chlamydomonas genome and divided them into 4 families. Using the amino acid sequences for known and putative PHTs from various land plants, green algae, diatoms and bacteria and the Neighbor-Joining method, we performed a phylogenetic analysis of the protein encoded by the *Cre09.g396950* gene (annotated as CrPHT4-9 or CrPHT4I at Phytozome ver. 13). As shown in Fig. 1, CrPHT4-9 shared the closest evolutionary history with the PHT4 family members from land plants, green algae and diatoms. More specifically, CrPHT4-9 belongs to the same clade as CrPHT4-7 and AtPHT4;5 (Fig. 1, Supplementary Fig. S1). A function in Pi transport was demonstrated for all Arabidopsis PHT4 proteins as well as for CrPHT4-7 when expressed heterologously in yeast (Guo et al. 2008; Tóth et al. 2024a). AtPHT4;1 functioned as a Pi transporter also when expressed in *Escherichia coli* (Pavon et al. 2008). In addition, AtPHT4;4 featured ascorbate transport activity in proteoliposomes (Miyaji et al. 2015).

The multiple sequence alignment (ClustalW 2.1) shown in Supplementary Fig. S1 reveals twelve conserved transmembrane domains in CrPHT4-9 (predicted by DeepTMHMM). Notably, this alignment also demonstrates a high conservation of residues that have been shown to be important for Pi transport in AtPHT4;1 (Ruiz-Pavon et al. 2010). These findings allow us to postulate an important function for CrPHT4-9 in cellular Pi and/or ascorbate transport.

### PHT4-9 is located in the thylakoid membrane and pyrenoid tubules and transports Pi in a yeast strain lacking Pi transporters

**Figure 2.**
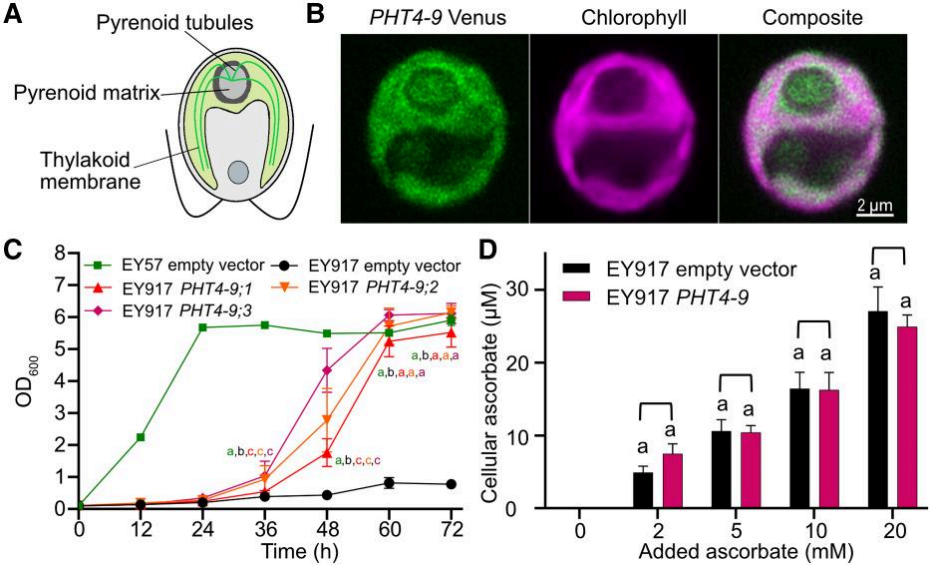
CrPHT4-9 is localized in the thylakoid membrane and improves growth of a yeast Pi transporter mutant. **A)** Schematic of the Chlamydomonas cell with the pyrenoid within the cup-shaped chloroplast. **B)** Representative confocal microscopic images of a Chlamydomonas WT cell expressing *CrPHT4-9*-Venus. The merged Venus and chlorophyll fluorescence image show localization to the chloroplast thylakoid membrane and the tubules traversing the pyrenoid. **C)** Growth of the Pi-transporter-deficient yeast strain EY917 expressing the empty vector or *CrPHT4-9* (3 independent transformants are shown) in comparison with the WT strain EY57 with empty vector. **D)** Uptake of ascorbate into yeast strain EY917 expressing *CrPHT4-9* in comparison to the control strain. The cultures were incubated with 0, 2, 5, 10, 20 mm ascorbate for 15 min. Data in **C)** and **D)** are means ± SEM of 4 biological replicates. Different letters indicate statistically significant differences among the strains (*P* < 0.05 using Tukey one-way ANOVA).

Wang et al. (2023) developed a PB-Chlamy localization prediction tool trained on over 1,000 candidate proteins and generated a chloroplast protein atlas. CrPHT4-9 was not among the experimentally verified proteins in the same study, but the presence of an N-terminal chloroplast targeting peptide was predicted by PB-Chlamy tool along with other available software (TargetP2.0 and LOCALIZER1.0.4) (Supplementary Fig. S1). Moreover, the Rubisco-binding motif WRXXL (Meyer et al. 2020) was found in the middle of the protein in a large hydrophilic sequence between the sixth and seventh transmembrane domains (Supplementary Fig. S1), suggesting co-localization with Rubisco inside the pyrenoid of the chloroplast (Fig. 2A).

To experimentally validate the predicted location, a construct of *CrPHT4-9* with the fluorescent marker Venus was generated and introduced into the Chlamydomonas wild-type (WT) strain CC-4533 (Emrich-Mills et al. 2021; Adler et al. 2024). As shown in the confocal images in Fig. 2B, the fluorescence signal was detected inside the chloroplast and extending into the pyrenoid. As CrPHT4-9 is predicted as a transmembrane protein (Supplementary Fig. S1), it is reasonable to conclude that it is more specifically localized in the thylakoid membrane network and tubules traversing the pyrenoid. While connected to the thylakoids, the tubules have a different protein enrichment (Franklin et al. 2024) and have been suggested to support the pyrenoid function in supplying CO_2_ to Rubisco located in the pyrenoid matrix (He et al. 2023).

To investigate whether CrPHT4-9 is a Pi transporter, we expressed it heterologously in the *Saccharomyces cerevisiae* strain EY917 that lacks 5 endogenous Pi transporters, namely PHO84, PHO87, PHO89, PHO90, and PHO91, grown in the absence of galactose that would otherwise enable the expression of PHO84 (Wykoff and O'Shea 2001). Recently, CrPHT4-7 was successfully expressed in this strain and demonstrated to function in Pi transport (Tóth et al. 2024a). Three independent *CrPHT4-9* transformants showed faster and considerably improved growth as compared with the parent EY917 strain (Fig. 2C). Although the transformants grew slower than the WT yeast strain EY57, the same levels were reached by the end of the experiment, thus strongly supporting Pi transport mediated by CrPHT4-9.

CrPHT4-9 also shares homology with AtPHT4;4 (Fig. 1, Supplementary Fig. S1), which was reported to facilitate ascorbate transport into the chloroplast (Miyaji et al. 2015). We performed uptake experiments in yeast in the presence of increasing concentrations of ascorbate. No statistically significant change in the cellular ascorbate content of EY917 transformed with *CrPHT4-9* was observed when compared with the parent strain (Fig. 2D), as has been reported in the case of CrPHT4-7 (Tóth et al. 2024a), suggesting that CrPHT4-9 is unlikely to transport ascorbate within the chloroplast. Taken together, these data indicate that CrPHT4-9 is located in the chloroplast thylakoid membranes and pyrenoid tubules, where likely functions as a Pi transporter.

### CrPHT4-9 is required for normal photoautotrophic growth under CO_2_-limiting conditions

**Figure 3.**
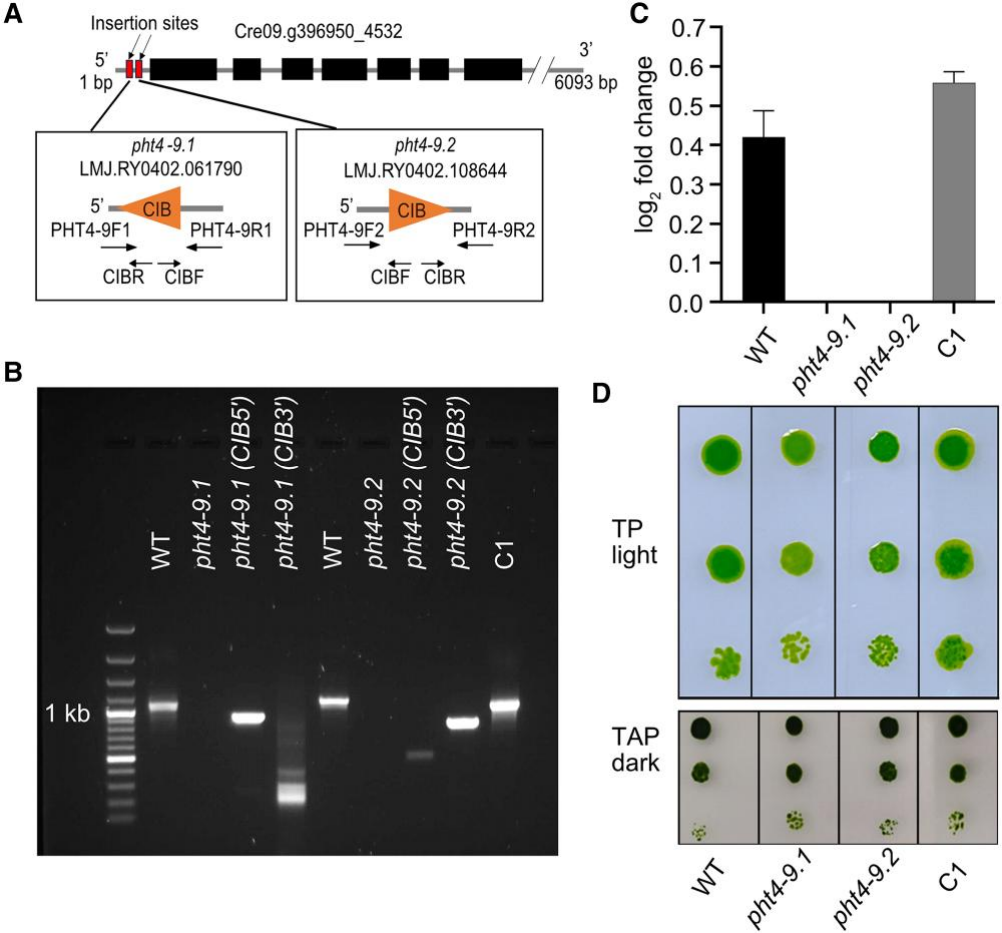
The *pht4-9* mutants exhibit defective photoautotrophic growth under CO_2_-limiting conditions rescued by genetic complementation. **A)** Physical map of *CrPHT4-9* (obtained from Phytozome ver. 13) indicating the position of the *CIB* cassette insertion in 2 independent *pht4-9.1 and pht4-9.2* mutants as well as the primers used for PCR amplification. **B)** PCR amplification of the *CrPHT4-9* locus in the genomic DNA from the WT, the *pht4-9.1* and *pht4-9.2* mutants (with no amplified product but a *CIB* cassette insertion), and the *pht4-9.1* mutant complemented with *CrPHT4-9* (C1 strain). **C)**  *CrPHT4-9* expression as determined by RT-qPCR in the 4 strains. Data are means ± SEM of 3 biological replicates. **D)** Spot growth test of WT, the *pht4-9.1, pht4-9.2* and complemented C1 strains. Chlamydomonas cells at a density of 5 × 10^5^ cells mL^−1^ were grown in a serial dilution (1, 1:10, and 1:100) with air levels of CO_2_ on agar plates with standard TP in the light at 100 *μ*mol photons m^−2^ s^−1^ or with TAP in the dark. While the *pht4-9* mutants grew similarly to WT on the control plate with TAP in the dark, they displayed reduced growth in TP in the light. The complemented C1 strain resembled WT in both conditions. The shown spot test is representative of 5 different experiments.

Two independent *pht4-9* mutant strains were obtained from CLiP (Li et al. 2019). The LMJ.RY0402.061790 strain (hereafter named *pht4-9.1*) and the LMJ.RY0402.108644 (*pht4-9.2*) had the *CIB1* cassette inserted at the expected position in the 5`-UTR of the *CrPHT4-9* gene sequence (Fig. 3, A and B). To obtain a complemented mutant strain, we generated a construct of *CrPHT4-9* with a mScarlet-I C-terminal tag (Emrich-Mills et al. 2021) and introduced it into the *pht4-9.1* mutant. The presence of mScarlet-I was predominantly used to screen for positive transformants in the growth phenotyping experiment. Among the positive transformants, the complemented C1 strain was confirmed by PCR and RT-qPCR (Fig. 3, B and C). To test whether CrPHT4-9 deficiency impacts growth, we first performed semi-quantitative spot tests of WT and mutants on agar plates. When grown on minimal Tris-Phosphate (TP) medium in the light at 100 *μ*mol photons m^−2^ s^−1^ and air levels of CO_2_ (i.e. CO_2_-limiting conditions), the *pht4-9.1* and *pht4-9.2* mutants exhibited slightly retarded growth relative to WT, while no such effect was observed in the mutants grown on Tris-acetate-phosphate medium (TAP) in the dark (Fig. 3D). This result validates the photosynthetic phenotype of *pht4-9* mutants reported in the high-throughput screen of CLiP (Fauser et al. 2022). The complemented C1 strain displayed restored growth in spot tests on TP agar plates in the light (Fig. 3D), showing that CrPHT4-9 supports photoautotrophic growth.

**Figure 4.**
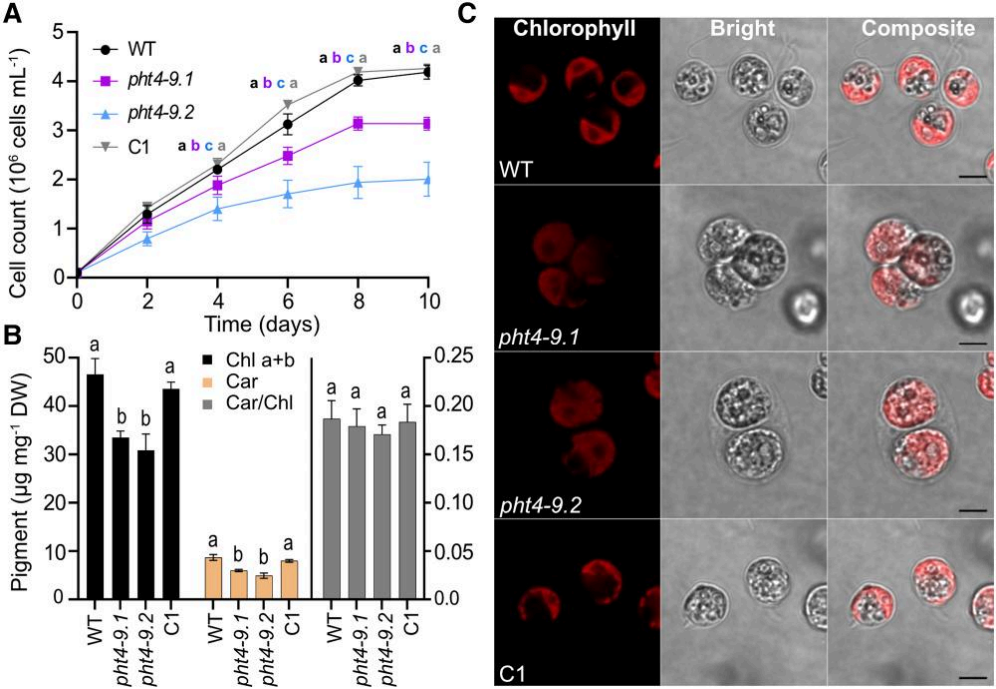
CrPHT4-9 is required for normal growth, photosynthetic pigment content and chloroplast ultrastructure under CO_2_-limiting conditions. Chlamydomonas cultures were grown in standard TP medium in the light at 100 *μ*mol photons m^−2^ s^−1^ and air levels of CO_2_. **A)** Growth in terms of cell number was monitored every 2 days. The *pht4-9* mutants grew slower and reached a lower cell number than WT and the complemented C1 strain. **B)** Total chlorophyll (Chl a + b) and carotenoid (Car) contents expressed per dry weight (DW), as well as Car/Chl ratio from 4-day-old cultures. The data in **A)** and **B)** are means ± SEM of 3 biological replicates. Different letters indicate statistically significant differences among the genotypes with *P* < 0.05 using Tukey one-way ANOVA. **C)** Ultrastructure of chloroplasts from 4-day-old cultures was visualized by confocal microscopy and chlorophyll fluorescence (scale bar 5 *μ*m). The *pht4-9* mutans displayed enlarged cells with altered chloroplast ultrastructure while the complemented C1 strain resembled WT.

When cultivated in liquid TP medium at air levels of CO_2_ and 100 *μ*mol photons m^−2^ s^−1^ light, the *pht4-9* mutants grew slower than WT (based on cell count per mL) especially toward the end of the 10-day experiment (Fig. 4A, Supplementary Table S1). The chlorophyll (Chl) and carotenoid contents expressed per dry weight were statistically significantly lower in the mutants, without however affecting the carotenoid/Chl ratio (Fig. 4B). The complemented C1 strain grew like WT for the entire duration of the experiment and had WT-like pigment content (Fig. 4, A and B, Supplementary Table S1). Interestingly, confocal microscopic inspection on Day 4 revealed enlarged *pht4-9* mutant cells and the formation of palmelloid-like clusters (Fig. 4C), likely as a stress response (Suwannachuen et al. 2023). Moreover, cell architecture and chloroplast ultrastructure were severely disrupted and Chl autofluorescence from thylakoids was spread out (Fig. 4C). Indeed, transmission electron microscopy revealed an expanded thylakoid membrane network with a swollen lumen occupying a substantial portion of the chloroplast (Supplementary Fig. S2). Complementation of the mutant strain with *CrPHT4-9* (C1) restored the WT-like cell size, cell and chloroplast architecture (Fig. 4C, Supplementary Fig. S2). Based on the statistically non-significant alteration in the expression of 4 cell division marker genes, it is unlikely that the enlarged cell size of *pht4-9* mutants is caused by impaired cell division (Supplementary Fig. S3).

### PHT4-9 impacts photosynthetic electron transport and photoprotection

**Figure 5.**
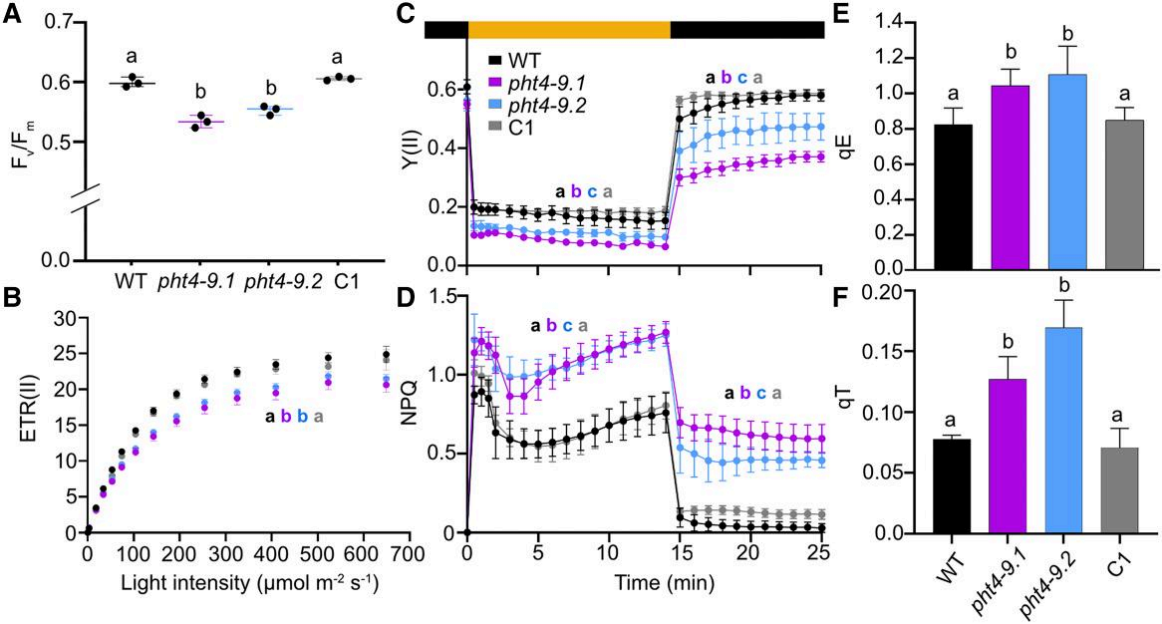
CrPHT4-9 is required for photosynthesis and photoprotection under CO_2_-limiting conditions. Chlamydomonas cultures were grown for 4 days in standard TP in the light at 100 *μ*mol photons m^−2^ s^−1^ and air levels of CO_2_. **A)** Photosystem II photochemistry (as assessed by *F_v_/F_m_*) was statistically significantly lower in the *pht4-9* mutants than in the WT and the complemented C1 strain. The plot shows the minimum, median and maximum values (as lines) together with the data for 3 biological replicates (as filled circles). **B)** Light response curves of photosystem II electron transport rate (ETR(II)) show reduced rates in the *pht4-9* mutants than in WT and C1 throughout the applied illumination range. **C)** Photosystem II efficiency (Y(II) in dark-adapted samples exposed to 325 *μ*mol photons m^−2^ s^−1^ illumination for 15 min followed by 10 min in the dark. **D)** Non-photochemical quenching (NPQ) obtained under identical conditions as in **C)**. While Y(II) was reduced, NPQ was enhanced in the *pht4-9* mutants as compared to WT and C1 throughout the treatment. **E)** The energy-dependent fast NPQ component (qE) as determined after 2 min of illumination at 325 *μ*mol photons m^−2^ s^−1^ (Supplementary Fig. S4A). **F)** The state transition-dependent NPQ component (qT) determined upon transition from red- to far-red light (Supplementary Fig. S5). Both qE and qT components were statistically significantly elevated in the *pht4-9* mutants as compared to WT and the C1 strain. The data presented in **B)** to **F)** are means ± SEM of 3 biological replicates. Different letters in **A)** to **F)** indicate statistically significant differences among the genotypes with *P* < 0.05 using Tukey one-way ANOVA.

The altered chloroplast ultrastructure and reduced growth in *pht4-9* prompted us to investigate the photochemical and non-photochemical reactions using induction kinetics of Chl *a* fluorescence in cultures grown in liquid TP medium in the light and air levels of CO_2_. The overall indicator of maximum photosynthetic performance (*F_v_*/*F_m_*) was slightly but statistically significantly lower in the *pht4-9* mutants than in WT and the complemented C1 strain (Fig. 5A). Rapid light response curves of electron transport rate in photosystem II (ETR(II)) also show lower levels in *pht4-9* mutants throughout the tested light intensity range (0 to 700 *μ*mol photons m^−2^ s^−1^, Fig. 5B).

Further, we analysed the slow kinetics of Chl *a* fluorescence in cells exposed to strong light (325 *µ*mol photons m^−2^ s^−1^) for 15 min followed by 10 min in the dark. The effective photosystem II activity (Y(II)) was lower in the *pht4-9* mutants throughout illumination and reached only about 60% of the WT level when the light was turned off (Fig. 5C), possibly indicating photoinhibition. A higher level of NPQ was induced in the *pht4-9* mutants in comparison with WT and the complemented C1 strain throughout the 15 min illumination (Fig. 5D). The relaxation kinetics during 10 min in the dark were also different, since the WT and the complemented C1 strain returned within approx. 6 min to minimal levels, while the *pht4-9* mutants retained a higher level of NPQ, indicating sustained quenching. The qE component of NPQ, estimated as the fraction of NPQ that is rapidly inducible in the first 2 min of light and reversible in the dark (Ruiz-Sola et al. 2023), was statistically significantly higher in the *pht4-9* mutants than in WT and the complemented C1 strain (Fig. 5E, Supplementary Fig. S4A), while Y(II) induced by a 2-min illumination, was similar among genotypes and returned to initial values (Supplementary Fig. S4B).

In addition to qE, state 1 to state 2 transition is another important strategy for Chlamydomonas to protect cells against high light. State transition involves the phosphorylation of light harvesting antennae resulting in detachment from PSII followed by aggregation in a quenched state and partial association to PSI (Allorent et al. 2013; Unlu et al. 2014; Erickson et al. 2015; Goldschmidt-Clermont and Bassi 2015). The state-transition-dependent quenching (qT) component cannot be clearly resolved in the NPQ kinetics in Fig. 5D. Therefore, we measured state transition using consecutive red and far-red illuminations (based on (Ruban and Johnson 2009; Tóth et al. 2024a)) and found that both *pht4-9* mutants exhibited higher qT than WT and the complemented C1 strain (Fig. 5F, Supplementary Fig. S5). Taken together, the low *F_v_/F_m_*, ETR(II) as well as the reduced Y(II) in high light and subsequent darkness (Fig. 5, A to C) suggest that the *pht4-9* mutants are impaired in photosynthetic electron transport and may suffer photodamage. Also, the increased qE and qT components of NPQ indicate enhanced photoprotective mechanisms in the *pht4-9* mutants and that qT was not limited by the lack of CrPHT4-9.

### PHT4-9 impacts the ATP synthase activity in the chloroplast but not the total ATP content

**Figure 6.**
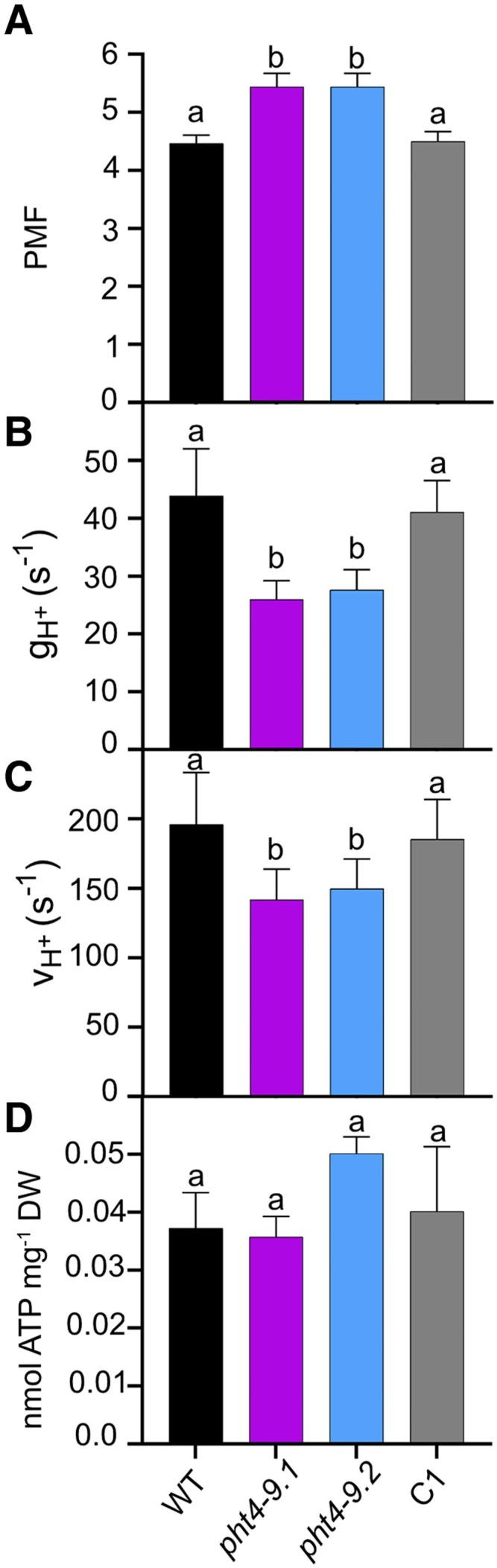
CrPHT4-9 modulates proton motive force and ATP synthase activity under CO_2_-limiting conditions. Chlamydomonas cultures were grown for 4 days in standard TP in the light at 100 *μ*mol photons m^−2^ s^−1^ and air levels of CO_2_, dark-adapted for 15 min, illuminated for 10 min at 660 *μ*mol photons m^−2^ s^−1^, followed by electrochromic shift decay measurements. The parameters were obtained at the end of the 10 min illumination and calculated as described in the Materials and methods section. Complete kinetics are shown in Supplementary Fig. S6. **A)** Proton motive force (PMF). **B)** H^+^ conductivity of the ATP synthase (g_H_^+^). **C)** H^+^ flux through the ATP synthase (v_H_^+^). While the PMF was statistically significantly elevated, the g_H_^+^ and ν_H_^+^ were reduced in the *pht4-9* mutants as compared to WT and the complemented C1 strain. **D)** Total ATP content was determined as described in Materials and methods and is expressed per dry weight. There were no statistically significant differences among the genotypes. The data are means ± SEM of 3 biological replicates. Different letters on top of bars indicate statistically significant differences among the genotypes with *P* < 0.05 using Tukey one-way ANOVA.

It was previously reported that Arabidopsis mutants lacking the thylakoid PHT4;1 transporter have reduced growth due to an altered Pi distribution in the chloroplast that reduces the activity of ATP synthase (Karlsson et al. 2015). Based on electrochromic shift experiments, we estimated the total PMF, the H^+^ conductivity (g_H_^+^) and the proton flux (v_H_^+^) through the thylakoid membrane in cells grown in TP medium for 4 days at 100 *μ*mol photons m^−2^ s^−1^ light at air levels of CO_2_. After 10 min of illumination, the PMF in the *pht4-9* mutants was slightly but statistically significantly higher than in WT (Fig. 6A). The g_H_^+^ was reduced to almost half of the WT value (Fig. 6B) and the overall ν_H_^+^ was also statistically significantly lower in the *pht4-9* mutants (Fig. 6C). Complementation with *CrPHT4-9* recovered PMF, g_H_^+^ and ν_H_^+^ to WT levels. The complete kinetics for the above-described parameters are provided in Supplementary Fig. S6.

Pre-treatment with N,N′-Dicyclohexylcarbodiimide (DCCD), an inhibitor of H^+^ translocation through the ATP synthase by modifying the c subunit (Joliot and Joliot 2001), statistically significantly reduced g_H_^+^ to a residual level in all strains (Supplementary Fig. S7). This indicates that the ATP synthase is the primary driver of the H^+^ efflux across the thylakoid membrane and that the observed differences in g_H_^+^ among untreated strains are likely due to varying ATP synthase activity.

Since the chloroplast is the main site of ATP production in Chlamydomonas, we next investigated if the absence of CrPHT4-9 in the thylakoid membrane affects ATP production by measuring ATP content. Cellular ATP concentration could not be calculated due to the challenge to accurately measure the cell volume, especially for the mutant cells forming clusters. Under standard growth conditions, the total ATP content expressed per dry weight showed no statistically significant differences among genotypes (Fig. 6D). In summary, these findings demonstrate that while the ATP synthase activity is diminished in the absence of CrPHT4-9, the total ATP content remains unaffected. This suggests that the mutants are less efficient in utilizing ATP for energy-dependent reactions within the chloroplast, such as CO_2_ assimilation.

### Low Pi and high CO_2_ restore cell morphology and diminish physiological differences

**Figure 7.**
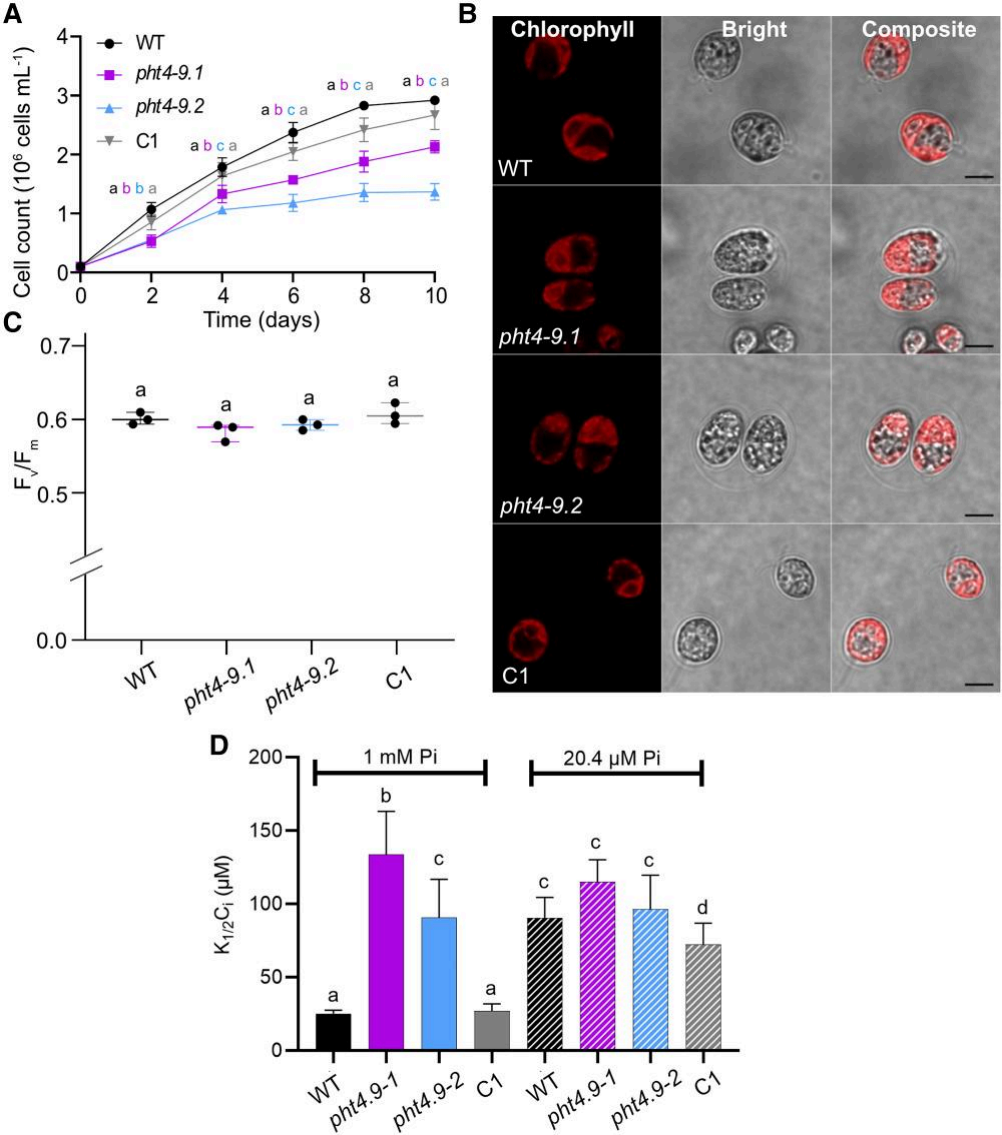
The growth phenotype of the *pht4-9* mutants is alleviated under Pi- and CO_2_-limiting conditions. Chlamydomonas cultures were grown in TP with 2% Pi (corresponding to 20.4 *µ*M final concentration of Pi) in the light at 100 *μ*mol photons m^−2^ s^−1^ and air levels of CO_2_. **A)** Growth in terms of cell number was monitored every 2 days. The *pht4-9* mutants grew slightly slower and reached a slightly but statistically significantly lower cell number than WT and the complemented C1 line. **B)** Ultrastructure of chloroplasts from 4-day-old cultures was visualized by confocal microscopy and chlorophyll fluorescence (scale bar 5 *μ*m). The *pht4-9* mutants and complemented C1 line displayed normal WT-like cell size and chloroplast structure. **C)** Photosystem II photochemistry (as assessed by *F_v_/F_m_*) was statistically not significantly different among the strains. The plot shows the minimum, median and maximum values (as lines) together with the data for 3 biological replicates (as filled circles). **D)** Half-saturation constant for inorganic carbon *K*_1/2_(*C_i_*) for oxygen-evolving activity at pH 7.4 was calculated from the O_2_ evolution versus *C_i_* curves presented in Supplementary Fig. S10, A and B. *K*_1/2_(*C_i_*) was statistically significantly higher for the *pht4-9* mutants as compared to WT and the C1 strain under Pi-replete conditions. *K*_1/2_(*C_i_*) did not differ among the strains under Pi-limiting conditions and the mutants under Pi-replete conditions. The data in **A)** and **D)** are means ± SEM of 3 biological replicates. Different letters indicate statistically significant differences among the genotypes with *P* < 0.05 using Tukey one-way ANOVA.

In order to investigate the relation to Pi supply of the observed growth differences between the mutants and the control strains (WT, C1) (Fig. 4A), we exposed the cultures to Pi- and CO_2_-limiting conditions. Growth in liquid medium containing 2% of Pi (20.4 *μ*M) in comparison with standard TP medium (1 mm Pi) resulted in diminished growth for all strains, particularly after 6 days of cultivation (cf. Fig. 4A and Fig. 7A; for growth rates, see Supplementary Table S1).

Interestingly, the cell size and chloroplast architecture of the *pht4-9* mutants appear to resemble the ones of WT and the C1 strains (Fig. 7B). Moreover, in cells grown in Pi- and CO_2_-limiting conditions, no statistically significant differences were observed among the genotypes in *F_v_/F_m_* (Fig. 7C), ETR(II) (Supplementary Fig. S8A), Y(II) and NPQ (Supplementary Fig. S8, B and C) as well as the qE and qT components of NPQ (Supplementary Fig. S8, D and E). When compared with the cells grown under Pi-replete conditions, obviously the photosynthetic performance of the mutants was improved (Supplementary Table S1). The PMF was also not statistically significantly different among the strains in low Pi, and the differences in g_H_^+^ and ν_H_^+^ were substantially reduced as compared with Pi-replete grown cells (Supplementary Fig. S9, A to C, Supplementary Table S1). These data indicate that low Pi supply alleviates rather than exacerbates the physiological effects of CrPHT4-9 deficiency. Interestingly, at low Pi, the mutants had a higher total ATP content as compared with WT (Supplementary Fig. S9D), but not statistically significantly different from the ATP content of the mutants in Pi-replete conditions (Supplementary Table S1). This suggests that the effects of CrPHT4-9 absence are not primarily related to stroma Pi availability.

CrPHT4-9 is located in the thylakoid membrane and the tubules traversing the pyrenoid (Fig. 2B), and the *pht4-9* mutants exhibit growth defects under CO_2_-limiting conditions (Fig. 3D). To investigate whether the observed effects in the *pht4-9* mutants are linked to the CCM, we measured photosynthetic O_2_ evolution across a range of external Ci concentrations. These measurements were conducted at pH 7.4 for cells grown for 4 days at air levels of CO_2_ and under either Pi-replete or Pi-limiting conditions (Supplementary Fig. S10, A and B). The determined half-saturation constant for inorganic carbon *K*_1/2_(*C_i_*) of approx. 25 *µ*M for the WT (CC-4533) strain was in the range characteristic of CO_2_-limited cultures (Ma et al. 2011; Mukherjee et al. 2019). Our results revealed that for cells grown at Pi-replete conditions, the *pht4-9* mutants exhibited statistically significantly higher *K*_1/2_(*C_i_*) values than the WT and the complemented C1 strain (Fig. 7D), suggesting a limitation in CCM activity. Under Pi-limiting conditions, nearly 3-fold increase in *K*_1/2_(*C_i_*) values was observed in WT and C1 strains, indicating a statistically significant limitation in CO_2_ fixation, consistent with Brooks (1986); Pieters et al. (2001). In these conditions, the absence of CrPHT4-9 had little additional impact on *K*_1/2_(*C_i_*) as compared with the Pi-replete conditions (Fig. 7D).

**Figure 8.**
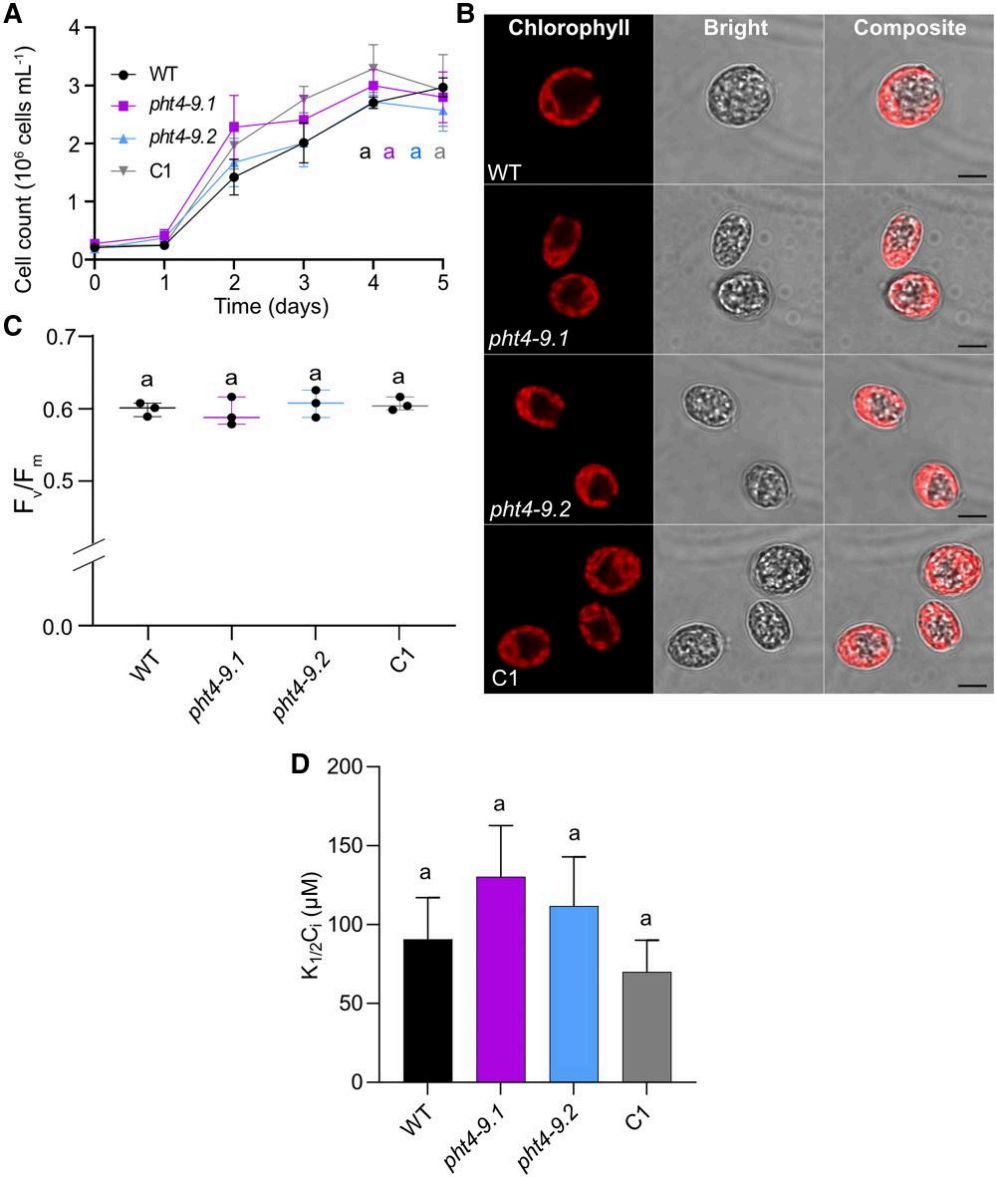
High CO_2_ conditions rescue the phenotype of *pht4-9* mutants. Chlamydomonas cultures were grown in standard TP in the light at 100 *μ*mol photons m^−2^ s^−1^ and air enriched with 2% CO_2_. **A)** Growth in terms of cell number was monitored every 2 days. The *pht4-9* mutants grew similarly to the WT and the complemented C1 strain. **B)** Ultrastructure of chloroplasts from 4-day-old cultures was visualized by confocal microscopy and chlorophyll fluorescence (scale bar 5 *μ*m). The *pht4-9* mutans and complemented C1 strain displayed normal WT-like cell size and chloroplast structure. **C)** Photosystem II photochemistry (as assessed by *F_v_/F_m_*) was statistically not significantly different among the genotypes. The plot shows the minimum, median and maximum values (as lines) together with the data for 3 biological replicates (as filled circles). **D)** Half-saturation constant for inorganic carbon *K*_1/2_(*C_i_*) for oxygen-evolving activity at pH 7.4 was calculated from the O_2_ evolution versus *C_i_* curves presented in Supplementary Fig. S10C. *K*_1/2_(*C_i_*) was not statistically significantly different among the genotypes. The data in **A)** and **D)** are means ± SEM of 3 biological replicates. Identical letters in **A)**, **C)** and **D)** indicate statistically non-significant differences among the genotypes with *P* > 0.05 using Tukey one-way ANOVA.

To further investigate the involvement of CrPHT4-9 in the CCM, we conducted CO_2_ supplementation experiments in a photobioreactor. Growth tests in liquid standard TP medium bubbled with air enriched with 2% CO_2_ revealed diminished growth differences among the strains (Fig. 8A, Supplementary Fig. S11). The cell size and chloroplast ultrastructure of the *pht4-9* mutants grown for 4 days appeared normal and resembled those of WT (Fig. 8B). Moreover, the *F_v_/F_m_* values for all strains at the same age were within the typical range of 0.6 observed for healthy WT cultures (Fig. 8C, Huang et al. (2023)). The determined *K*_1/2_(*C_i_*) of approx. 90 *µ*M for the WT at pH 7.4 was in the range characteristic of CO_2_-supplemented cultures (Ma et al. 2011). The difference in *K*_1/2_(*C_i_*) among the control lines and the *pht4-9* mutants was statistically insignificant (Fig. 8D, Supplementary Fig. S10C). These findings suggest that the *pht4-9* mutants may be limited in CO_2_ assimilation at air levels of CO_2_, leading to impaired photosynthetic electron transport, altered chloroplast structure, and ultimately reduced growth. Conversely, when the CCM is not required due to high CO_2_ levels, the absence of CrPHT4-9 no longer limits CO_2_ assimilation.

## Discussion

The impact of Pi on photosynthesis is complex and multifaceted, involving changes in the synthesis of pigments and membrane lipids, activation of enzymes, and production of energy. Pi homeostasis in the chloroplast of the model plant Arabidopsis is maintained by transporters from the pPT, PHT2 and PHT4 families, whose identities in algae are largely unknown (Marchand et al. 2018; Wang et al. 2020). Nevertheless, the envelope-localized TPT3 and PHT4-7 were characterized in Chlamydomonas and found important for photosynthesis and cell metabolism impacting growth (Huang et al. 2023; Tóth et al. 2024a). Studying the mechanism of Pi transport is crucial because in natural habitats as well as in industrial photobioreactors microalgae frequently encounter fluctuations in CO_2_ and Pi availability as well as changes in light intensity (Falkowski and Raven 1997; Perin and Morosinotto 2023). Consequently, understanding how chloroplast Pi homeostasis is regulated and its effects on photosynthesis is essential. Moreover, algae have capacity to accumulate substantial amounts of Pi (Sanz-Luque et al. 2020), and thus provides opportunities for biotechnological applications, such as production of Pi-rich fertilizers after recovery from wastewater (Slocombe et al. 2020). In the present study, we localize CrPHT4-9 to the thylakoid membrane network and the tubules traversing the pyrenoid, and provide multiple evidence for its importance in maintaining chloroplast Pi homeostasis and modulating photosynthesis.

Our sequence analysis identified CrPHT4-9 as a member of the major facilitator superfamily and closely related to the chloroplast-localized AtPHT4;1, AtPHT4;4, and AtPHT4;5 (Fig. 1). The presence of a predicted chloroplast targeting peptide and a putative Rubisco-binding motif (Supplementary Fig. S1), combined with the localization of the Venus-tagged *CrPHT4-9* construct to the chloroplast and its pyrenoid (Fig. 2, A and B) demonstrate that the thylakoid membrane and pyrenoid-traversing tubules are the most likely locations for CrPHT4-9. Meyer et al. (2020) demonstrated the crucial role of Rubisco-binding motifs in targeting of proteins to the pyrenoid and proposed that proteins harboring such motifs may anchor the Rubisco matrix to either the tubule membranes or to the starch sheath. Given the single Rubisco-binding motif, located within a large and disordered loop in the middle of the CrPHT4-9 protein (Supplementary Fig. S1) and the observed pyrenoid targeting, it is likely that this motif primarily facilitates targeting to the pyrenoid tubules rather than playing an important structural function. A similar targeting role for the Rubisco-binding motifs has been recently demonstrated for bestrophin-like protein 4, which is found at the center of the pyrenoid tubule network (Adler et al. 2024).

We demonstrated that CrPHT4-9 complements the growth defect of the yeast strain EY917 that lacks 5 endogenous Pi transporters reaching similar levels as the WT EY57 (Fig. 2C). While we did not perform a direct Pi transport assay in this study, the robust complementation of the yeast growth defect, combined with the EY917 strain's probable inability to import ascorbate (Fig. 2D), strongly support a Pi transport function for CrPHT4-9. Further experiments, for instance, employing proteoliposomes containing purified CrPHT4-9 could provide further confirmation of this function.

The cells of mutants lacking CrPHT4-9 formed clusters, were enlarged with disturbed chloroplast ultrastructure and exhibited retarded photoautotrophic growth under CO_2_-limiting and Pi-replete conditions, a phenotype that was reversed by genetic complementation (Figs. 3 and 4). The formation of palmelloid-like cell clusters is a mechanism essential for high light tolerance (Suwannachuen et al. 2023). Moreover, extensive thylakoid membrane swelling was previously reported for Chlamydomonas cells experiencing photoinhibition caused by high membrane energization and acidification of the thylakoid lumen (Topf et al. 1992). Also, mutation of VIPP1 caused extreme thylakoid swelling in Chlamydomonas (Nordhues et al. 2012). In our study, in addition to the extensive thylakoid swelling, photosynthetic electron transport was diminished in the *pht4-9* mutants, while NPQ and its components (the pH-dependent qE and the state-transition-dependent qT) were increased (Fig. 5). PMF, representing the membrane energization, increased in the *pht4-9* mutants, the ATP synthase activity decreased, while the total ATP content remained unaffected (Fig. 6). In addition, we found that the CCM was impaired in the *pht4-9* mutants (Fig. 7D).

Under Pi- and CO_2_-limiting conditions, the differences in chloroplast ultrastructure and photosynthetic parameters between WT and the *pht4-9* mutants were diminished as compared with Pi-replete conditions (Fig. 7, Supplementary Figs. S8, A to C, S9, A to C and Supplementary Table S1). The total ATP content of the mutants was higher than in WT in low Pi but unaffected as compared with Pi-replete conditions for all genotypes (Supplementary Fig. S9D and Supplementary Table S1). Our data on the WT agree with earlier findings that when Chlamydomonas cells are grown under Pi-limiting conditions, the photosynthetic activity and growth rates diminish along with decreased ATP synthesis (Moseley et al. 2006; Plaxton and Tran 2011). The mitigated phenotype of the *pht4-9* mutants under Pi-limiting conditions can be explained by a downregulation of the CCM and the CBB cycle. This leads to reduced ATP consumption and, consequently, a lower demand for Pi in the *pht4-9* mutants.

The CCM is known to facilitate CO_2_ delivery to Rubisco within the pyrenoid under CO_2_-limiting conditions, while it becomes unnecessary in high CO_2_, leading to pyrenoid disassembly and Rubisco relocation to the chloroplast stroma (He et al. 2023). Indeed, CO_2_ supplementation mitigated the effects of the absence of CrPHT4-9 as evidenced from improvements in chloroplast ultrastructure, cell growth and PSII efficiency (Fig. 8) as compared to CO_2_-limiting conditions (Figs. 4 and 5A). Consequently, the role of CrPHT4-9 appears less critical under high CO_2_ conditions.

The CO_2_-dependent phenotype of the *pht4-9* mutants demonstrates a connection between CPHT4-9 function and the CCM under CO_2_-limiting conditions. We suggest that other chloroplast Pi transporters may be involved under high CO_2_ or Pi-limiting conditions. Wang et al. (2020) predicted at least 5 other CrPHT4 members as chloroplast targeted. In the same study, the expression of *CrPHT4-3*, *CrPHT4-4*, and *CrPHT4-6* genes, all 3 coding for chloroplast-predicted proteins, was indeed statistically significantly affected by Pi starvation. In addition, other high-affinity Pi transporters, such as those belonging to the PTB family, have been found upregulated under Pi-limiting conditions (Moseley et al. 2006). Notably, CrPHT4-7 was localized to the chloroplast despite lacking a predicted targeting peptide for this organelle (Tóth et al. 2024a). Mutants lacking CrPHT4-7 displayed regular chloroplast morphology, diminished ATP levels and qT, that were both attributed to limited Pi availability in the chloroplast stroma (Tóth et al. 2024a). A comprehensive characterization of the chloroplast Pi transportome will elucidate the mechanism underlying chloroplast Pi homeostasis under Pi-limiting conditions.

The malfunction of CCM in the *pht4-9* mutants could be attributed to additional ATP demands to those for driving the CBB cycle in the chloroplast stroma. Since the linear electron transport produces less ATP than required for CO_2_ fixation, alternative mechanisms involving cyclic electron transport and import of mitochondrial ATP into the chloroplast supply the energy required for driving CCM (Burlacot et al. 2022) and the entire CBB cycle (Peltier et al. 2024). However, it is important to note that various chloroplast compartments also engage in protein synthesis, degradation, and phosphorylation, which are additional ATP-consuming processes.

The primary function of the pyrenoid is to enhance the efficiency of photosynthesis, particularly in environments with low CO_2_ levels. This organelle is primarily composed of densely packaged Rubisco and the linker protein EPYC1, with a surrounding starch sheath that acts as a diffusion barrier. The tubules traversing the pyrenoid matrix are crucial for supplying the pyrenoid with necessary components from the chloroplast stroma, as the 2 compartments are not continuous (Catherall et al. 2025). Pyrenoid function relies on both Pi and ATP. A comprehensive proteomic study by Mackinder et al. (2017) revealed that the pyrenoid tubules contain several subunits of PSI, PSII, cytochrome b_6_/f and ATP synthase. These subunits likely represent assembly intermediates or inactive complexes undergoing repair, which may require Pi and ATP. In a subsequent study, Zhan et al. (2018) identified a few PSI, PSII and light harvesting subunits, but no ATP synthase or cytochrome b_6_f subunits were detected. Additionally, state transitions, which are dependent on protein phosphorylation/dephosphorylation, have been observed in the pyrenoids, although they are less pronounced as compared to those in stromal thylakoids (Zhang et al. 2022).

The localization and/or activation of several key pyrenoid proteins (*e.g*. EPYC1, CAH3) are regulated by phosphorylation and CO_2_ availability, thereby impacting CO_2_ assimilation by Rubisco (He et al. 2023). These processes may potentially involve ATP-binding/hydrolysing proteins like an ABC transporter that interacts with the small subunit of Rubisco (Mackinder et al. 2017), and the ribosomal-associated ABCF6 protein involved in RNA metabolism (Lau et al. 2023). Furthermore, the alpha-type carbonic anhydrase CAH3 exhibits a dynamic localization between the thylakoid lumen and the pyrenoid tubule lumen, influenced by CO_2_ levels and phosphorylation (Blanco-Rivero et al. 2012).

Dephosphorylation processes may also occur in the pyrenoid matrix, as evidenced by the localization of xylulose-1,5-biphosphate (XuBP) phosphatase (Wang et al. 2023). The Rubisco linker protein EPYC1, previously shown to be phosphorylated under CO_2_-limiting conditions in Chlamydomonas (Turkina et al. 2006), is located in the pyrenoid matrix and essential for its assembly (He et al. 2020). Although a potential kinase was identified in a CCM interactome (Mackinder et al. 2017), the role of EPYC1 phosphorylation/dephosphorylation in pyrenoid function remains unclear.

The observed examples of protein phosphorylation/dephosphorylation within the pyrenoid support an active Pi metabolism that extends into the thylakoid membrane given their continuous lumen (He et al. 2023). We propose that CrPHT4-9 transports the Pi resulting from ATP-dependent reactions in the pyrenoid matrix into the tubule lumen, thereby indirectly supporting CCM activity. We further hypothesize that Pi accumulated in the thylakoid lumen is subsequently transported into the chloroplast stroma via CrPHT4-9, potentially to support ATP synthesis, as proposed for the thylakoid-localized PHT4;1 in Arabidopsis (Pavon et al. 2008; Karlsson et al. 2015). The *pht4;1* mutants exhibit a similar photosynthetic phenotype to *pht4-9* mutants, that is enhanced NPQ, reduced g_H_^+^, ν_H_^+^, and plant growth. These phenotypes are attributed to excess H^+^ in the thylakoid lumen and limited Pi availability for the ATP synthase (Karlsson et al. 2015). Unlike in the *pht4;1* mutants, the PSII activity parameters (*F_v_/F_m_*, Y(II), ETR (II)) were diminished in the *pht4-9* mutants and the chloroplast ultrastructure was severely altered, suggesting that the dual localization of CrPHT4-9 in the thylakoid membrane and pyrenoid tubules contributes to the severe phenotype observed. PHT4 proteins facilitate Pi transport in symport with H^+^ and/or Na^+^ (Versaw and Harrison 2002; Guo et al. 2008; Pavon et al. 2008). As described above for AtPHT4;1, CrPHT4-9 likely uses the H^+^ gradient generated by the electron transport across the thylakoid membrane as a driving force for transporting Pi out to the stroma. We have increasing knowledge about the protein composition and processes occurring in the pyrenoid matrix and tubules (Lau et al. 2023; Wang et al. 2023; Catherall et al. 2025), but no data are available about either their pH or Pi concentration. Further experimental evidence is required to elucidate the specific transport mechanism of CrPHT4-9 across the pyrenoid tubules.

In summary, our findings highlight the role of CrPHT4-9 as a thylakoid- and pyrenoid-localized Pi transporter, which is used to modulate photosynthesis, thereby affecting chloroplast ultrastructure and growth. More broadly, our study contributes to expanding the pyrenoid protein atlas (Wang et al. 2023) and identifying potential targets for biotechnological strategies to improve photosynthesis in green algae and crop plants.

## Materials and methods

### Strains and culture conditions

Chlamydomonas WT strain CC-4533 and the *pht4-9* mutant strains LMJ.RY0402.061790 (*pht4-9.1*) and LMJ.RY0402.108644 (*pht4-9.2*) were obtained from the CLiP library at the Chlamydomonas Resource Centre (https://www.chlamylibrary.org/). The strains were maintained in the dark on agar plates (1.2% w/v) prepared with Tris-Acetate-Phosphate plates (TAP, containing 1 mm potassium-phosphate, 1.5% w/v agar).

To confirm the *CIB1* cassette insertion in the *pht4-9* mutants, genomic DNA was extracted from WT and mutants, and PCR was performed with the appropriate combination of primers (Supplementary Table S2). Cell count was determined using a hemocytometer or a Luna-FL dual fluorescence cell counter (Logos Biosystems Inc.).

For the spot tests, a loopful of culture was transferred to TAP medium and grown in the dark for 3 days. The cells were then resuspended to a density of 5 × 10^5^ cells mL^−1^ in TAP, spotted at different dilutions (1, 1:10 and 1:100) on TAP plates, and allowed to grow in the dark at 23 °C. Alternatively, cells were resuspended in TP medium at the same cell density as above and spotted onto TP plates at different dilutions (1, 1:10 and 1:100), at 23 °C in continuous light (100 *µ*mol photons m^−2^ s^−1^).

For growth analysis, cells were resuspended at a cell density of 1 × 10^6^ cells mL^−1^ in standard TP (containing 1 mm Pi), or TP with 2% Pi (corresponding to 20.4 *µ*M final concentration of Pi) and air levels of CO_2_ in a volume of 50 mL in Erlenmeyer flasks. For photosynthetic analyses, strains were grown in liquid TP medium at an initial density of 1 × 10^6^ cells mL^−1^ for 4 days. For the growth assays in high CO_2_, the cells were first cultured in TAP in Erlenmeyer flasks for 3 days before being placed in a Multi-Cultivator MC 1000-OD instrument (Photon System Instruments, Brno, Czech Republic). The cultures were grown at 23 °C for 4 days in TP bubbled with air enriched with 2% CO_2_ in continuous light (100 *µ*mol photons m^−2^ s^−1^). After 4 days of cultivation in flasks at air levels of CO_2_ or in the Multi-Cultivator with 2% CO_2_, the pH dropped to approx. 4.5 and 4.0, respectively. The drop in pH did not, however, impact cell viability, as indicated by the photosynthetic parameter *F_v_/F_m_* (Figs. 5A, 7C and 8C).

### Sequence analyses and phylogeny

The nucleotide and amino acid sequences of CrPHT4-9 (Cre09.g396950) and other potential Pi transporters were obtained from Phytozome (ver. 13, https://phytozome-next.jgi.doe.gov/) and from Wang et al. (2023). Pi transporter protein sequences from Arabidopsis were obtained from the plant membrane protein database Aramemnon (https://aramemnon.botanik.uni–koeln.de/). Other representative PHT4 protein sequences in *Chlorella zofingiensis, Chlorella vulgaris, Volvox carteri* (green alga); *Oryza sativa, Zea mays* (vascular plants); *Cyanidioschyzon merolae* (red alga); *Phaeodactylum tricornutum, Thalassiosira pseudonana* (diatoms); and *E. coli* were obtained from NCBI (https://www.ncbi.nlm.nih.gov/) or Phytozome using CrPHT4-9 as query. The amino acid sequences were aligned using ClustalW 2.1 in MEGA11 (Tamura et al. 2021), and the phylogenetic tree was generated using the Neighbor-Joining method (Saitou and Nei 1987) with 500 bootstraps and the default settings of MEGA11. The evolutionary distances were computed using the Poisson correction method (Zuckerkandl and Pauling 1965) and expressed as the number of amino acid substitutions per site. This analysis involved 58 amino acid sequences. All ambiguous positions were removed for each sequence pair (pairwise deletion option). There were a total of 2,784 positions in the final dataset. TMDs were predicted using DeepTHMMM (https://dtu.biolib.com/DeepTMHMM) and chloroplast targeting sequence using TargetP2.0 (https://services.healthtech.dtu.dk/services/TargetP-2.0/) and LOCALIZER1.0.4 (https://localizer.csiro.au/).

### Chlamydomonas transformation for localization and mutant complementation

The plasmids to generate the fluorescent-tagged *CrPHT4-9* and the complemented *pht4-9.1* mutant were prepared employing the recombineering method described previously (Emrich-Mills et al. 2021) using primers and *CrPHT4-9* homology arm specified in Supplementary Table S1. The recombineering plasmids contained either hygromycin AphVII (pLM161) or paromomycin AphVIII (pLM099) selection markers and are available through Chlamydomonas Resource Centre. *CrPHT4-9* was expressed under the native promoter with a 3 × FLAG and either a Venus or an mScarlet-I C-terminal tag (specified). Both fluorophores have been previously established for protein localization in the Chlamydomonas chloroplast (Emrich-Mills et al. 2021; Adler et al. 2024) and are clearly distinguishable from chlorophyll autofluorescence using our specified excitation and emission filters, described below.

For Chlamydomonas transformation, 29 ng kbp^−1^ of plasmid was linearized by incubating with I-SceI for 1 h and 37 ^o^C. The reaction was inactivated at 65 °C for 20 min. Chlamydomonas cells were grown in TAP, illuminated at 20 to 40 *μ*mol photons m^−2^ s^−1^ to a density of 2 to 4 × 10^6^ cell mL^−1^. The cells were harvested by centrifugation at 1000 × *g* for 10 min and resuspended in TAP with 40 mm sucrose at a concentration 2 to 4 × 10^8^ cell mL^−1^. TAP sucrose and electroporation cuvettes were chilled on ice prior to use. Immediately prior to electroporation by NEPA21 (NEPAGENE), 112 *μ*L of cells and 8 *μ*L of linearized plasmid were added to a 1 mm electroporation cuvette. Electroporation conditions are described in Supplementary Table S3. Cells were immediately recovered in TAP with 40 mm sucrose and incubated overnight at 21 °C in the dark with gentle shaking. Cells were subsequently transferred onto TAP agar plates with paromomycin (20 *μ*g mL^−1^) or hygromycin (25 *μ*g mL^−1^) for 1 to 2 weeks while illuminated at 20 *μ*mol photons m^−2^ s^−1^, to allow screening for growth. Single colonies of positive transformants were genotyped by PCR to screen for successfully complemented transformants using one primer located in the *CrPHT4-9* gene and one in the C-terminal tag. Further confirmation of the complemented strain was obtained by PCR and RT-qPCR with *CrPHT4-9* specific primers (Supplementary Table S2).

### Confocal microscopy

For WT and the *pht4-9.1* strains fluorescently labeled with either Venus or mScarlet-I fluorescent tag, respectively, positive transformants were directly screened for fluorescence using a Typhoon scanner (GE Healthcare, San Diego, CA, USA) and then picked and prepared for further imaging. Cells were grown in TAP, illuminated at 20 to 40 *μ*mol photons m^−2^ s^−1^ to a density of 1 × 10^6^ cell mL^−1^ and then transferred to TP media for 24 h prior to imaging. Cells were imaged in Ibidi 8-well slides overlaid by TP with 1.5% low melting point agarose to prevent cell mobility. Images were acquired using a Zeiss LSM880 confocal microscope using a 63 × 1.40 NA oil planapochromat lens. Images were analyzed using ZEN 2.1 software (Zeiss, San Diego, CA, USA) and FIJI. For Venus-tagged lines: Venus was excited using 514 nm laser, at 4% intensity, the emission was collected using 525 to 550 nm filters, and master and digital gains of 790 and 4.0, respectively. The corresponding chlorophyll signal was excited using a 561 nm laser at 1.2% intensity, the emission was collected using 665 to 705 nm filters, and master and digital gains of 720 and 2.0, respectively. For the mScarlet-I tagged lines: mScarlet-I was excited using a 514 nm laser at 2% intensity, the emission was collected using 580 to 600 nm filters, and master and digital gains of 700 and 2.8, respectively. The corresponding chlorophyll signal was also excited using a 561 nm laser at 2% intensity; however, the emission was collected using 665 to 705 nm filters, and master and digital gains of 550 and 1.0, respectively.

Cell imaging with chlorophyll autofluorescence was performed using an Olympus Fluoview FV1000 microscope (Olympus Life Science Europa GmbH, Hamburg, Germany). Samples were prepared for imaging on clean glass coverslips (Menzel-Gläser, 24 × 40 mm, Thermo Scientific), and wiped with 100% ethanol, to increase surface tension for better adhesion of the cells. The cells were immobilized by suspending them in 0.4% low melting point agarose. Single optical sections or successive 3D optical sections of the cells were taken using the UPLSAPO 60× (NA: 1.35) oil immersion objective. Microscope configuration was as follows: sampling speed: 4 μs/pixel; line averaging: 2×; scanning mode: unidirectional; zoom: 7×; excitation for chlorophyll fluorescence: 543 nm; maximum laser transmissivity value: 40%. Chlorophyll autofluorescence was detected between 650 to 750 nm. Images were pseudo colored and analyzed using Olympus Fluoview software (version 4.0.3.4) and ImageJ (version 1.52a).

### Reverse-transcriptase quantitative PCR analysis

RNA was extracted from cells at Day 0 and Day 4 of growth as described above using Trizol reagent following the method provided by Invitrogen, and DNA was removed with E1091 DNAse (Omega Bio-Tek). 500 ng of RNA per sample was used as template for cDNA, which was synthesized using iScript cDNA synthesis Kit (Bio-Rad, Hercules, CA, USA). Reverse-transcriptase quantitative PCR (RT-qPCR) analyses were conducted with a SsoAdvanced Universal SYBR Green Supermix on a CFX96 Touch Thermal Cycler (Bio-Rad). RT-qPCR amplifications were done at the following conditions: initial denaturation for 2 min at 95 °C, followed by 40 cycles of denaturation for 5 s at 95 °C, annealing for 30 s at 60 °C and extension for 10 s at 72 °C. After amplification, melt-curve analyses were performed. Gene-specific primers were used for *CrPHT4-9* and 4 cell division markers (Supplementary Table S2). ΔCq method (ΔΔCt) was used to calculate relative expression in 3 biological replicates using genes encoding G-protein subunit-like protein (GBLP) (Schloss 1990) and β-tubulin as endogenous controls.

### Complementation of *Saccharomyces cerevisiae* EY917 mutant with *CrPHT4-9*

For heterologous expression, we used the *S. cerevisiae* WT strain EY57 (*MATa ade2-1 trp1-1 can1-100 leu2-3,112 his3-11,15 ura3*) and the mutant strain EY917 in which 5 Pi transporters were inactivated *(Δpho84 Δpho87 Δpho89 Δpho90 Δpho91*) (*MATα ade2-1 trp1-1 can1-100 leu2-3,112 his3-11,15 ura3 pho84::HIS3 pho87::CgHIS3 pho89::CgHIS3 pho90::CgHIS3 pho91::ADE2, pGAL1-PHO84* (EB1280) (Wykoff and O'Shea 2001). The *GAL1* promoter drives the expression of *PHO84* enabling growth on galactose-containing media (Wykoff and O’Shea 2001). The *CrPHT4-9* coding sequence excluding the chloroplast transit sequence (amino acids 1 to 82) and codon optimized for yeast (synthesized by GeneArt, Life Technologies, Carlsbad, CA, USA) with *BamHI* and *EcoRI* restriction sites at the 5′ and 3′ ends was cloned into the similarly digested vector p426-TEF (containing *URA3* marker), generating the transformation plasmid. We transformed the EY917 strain with the plasmid containing *CrPHT4-9* by selecting for the *URA3* marker. We followed the transformation protocol by Gietz and Schiestl (2007). For transformation, strains were grown in synthetic media lacking uracil and containing 2% galactose. Growth of the strains expressing the empty vector or *CrPHT4-9* was monitored using OD_600_ every 12 h for up to 3 days, as described by Tóth et al. (2024a).

### Ascorbate uptake in *S. cerevisiae*

The *S. cerevisiae* mutant strain EY917 expressing *CrPHT4-9* was used to perform ascorbate uptake as described previously (Tóth et al. 2024a). Briefly, log phase cultures grown in yeast synthetic media with 2% glucose (w/v) and appropriate amino acids on a rotatory shaker at 30 °C for 24 h were taken and resuspended at OD_600_ of 0.8. The cells were treated with various concentrations of ascorbate (0, 2, 5, 10, and 20 mm) for 15 min at 30 °C with shaking (120 rpm), centrifuged, washed thrice, and the pellet was frozen in liquid nitrogen until use. Cells were broken by vortexing for 30 s with glass beads (425 to 600 *µ*m, Sigma-Aldrich, St. Louis, USA) and the ascorbate content was determined as described by Kovács et al. (2016).

### ATP content determination

ATP was measured using the Adenosine 5′-triphosphate (ATP) Bioluminescent Assay Kit (Sigma-Aldrich) according to the instructions of the manufacturer. 3 × 10^7^ algal cells were harvested by centrifugation (21,130 × *g*, 1 min, 4 °C) and washed once with ice cold sterile water. The pellets were resuspended in 250 *µ*L ice cold sterile water. Cells were broken by vortexing for 2 min with 80 *µ*L quartz sand. After the vortexing, the samples were centrifuged (21,130 × *g*, 1 min, 4 °C). 200 *µ*L of the supernatant were transferred to EZ-10 Spin Columns (Bio Basic Inc.) and rapidly spun down (21,130 × *g*, 1 min, 4 °C). Until ATP determination, the samples were stored on ice.

### Pigment content determination

Chlamydomonas cells were grown for 4 days in TP at 100 *μ*mol photons m^−2^ s^−1^. Cells were harvested by centrifugation and the chlorophyll (Chl a + b) and carotenoids (Car) were extracted in 100% (v/v) methanol. Pigment concentrations were determined according to Lichtenthaler and Wellburn (1983). Specific volumes of cells normalized to Chl content were lyophilized to determine the pigment content per dry weight.

### Chlorophyll *a* fluorescence measurements

Chlorophyll fluorescence was recorded using DUAL-PAM 100 instrument (Heinz Walz GmbH) for cells grown in TP as described above. Before the measurement, cells were resuspended to 30 *µ*g Chl mL^−1^ and incubated on a rotary shaker at 50 rpm in the dark for 15 min before recording Chl fluorescence. A 3,000 *µ*mol photons m^−2^ s^−1^ saturating pulse was applied to the samples in a cuvette under continuous stirring for determination of the maximal fluorescence yield in the dark state (F_m_) and maximal fluorescence yield during the period with actinic light (F_m_′). PSII photochemistry was assessed by the *F_v_/F_m_* parameter (Genty et al. 1989). For measurements involving longer illumination, cells were filtered onto a GF/C filter equivalent to 30 *µ*g Chl mL^−1^ that was placed between 2 microscopy coverslips with a spacer to allow for gas exchange. ETR was determined from rapid light curves using light intensities ranging from 26 to 700 *μ*mol photons m^−2^ s^−1^. NPQ was determined from slow kinetics during actinic illumination at 325 *μ*mol photons m^−2^ s^−1^ for 15 min followed by 10 min of dark relaxation. NPQ and Y(II) were calculated based on changes in fluorescence as (*F_m_–F_m_*′)/*F_m_*′ and (*F_m_*′–*F*)/*F_m_*′ (Genty et al. 1989). qE was calculated as fraction of NPQ that is rapidly inducible in the light and reversible in the dark (Ruiz-Sola et al. 2023).

For analyzing state transition, actinic red light (AL, 15 *μ*mol photons m^−2^ s^−1^) and far-red (FR) light (255 *μ*mol photons m^−2^ s^−1^) were employed for 15 min (phase 1) on dark-adapted cultures. After this phase, the far-red light was turned off and only red-light illumination was employed for 15 min to induce state II (phase 2). Finally, we used again the red light–far-red light combination for 15 min to drive the state II—state I transition (phase 3). During the measurement, saturating light pulses (8000 *μ*mol photons m^−2^ s^−1^ for 600 ms) were given every minute. qT parameter was calculated as: qT = (F_m_^I^—F_m_^II^)/F_m_^II^, in which F_m_^I^ was determined at the end of the phase 3, and F_m_^II^ at the end of phase 2.

### Inorganic carbon concentration-dependent photosynthetic O_2_ evolution measurements

Photosynthetic O_2_ evolution was measured using a PreSens OXY-1 SMA instrument equipped with Oxygen dipping probe DP-PSt7 (PreSens Precision Sensing GmbH, Germany) and controlled by a PreSens Measurement Studio PMS2 software. Cultures were grown for 4 days in liquid TP medium as described above. Cells were then pelleted and resuspended in HEPES-NaOH buffer (pH 7.4) in air sealed 15-mL serum bottles, that had been previously bubbled with N_2_ to remove dissolved CO_2._ The Chl concentration was set at 15 *µ*g Chl mL^−1^. The PreSens probe was inserted in the culture, and the cells were illuminated at 300 *µ*mol photons m^−2^ s^−1^ to deplete the dissolved inorganic carbon (Ci) until net O_2_ evolution ceased. Increasing concentrations (0 to 5 mm) of NaHCO_3_ solutions were injected into the buffer. The half-saturation constant for *C_i_* (*K*_1/2_(*C_i_*)) was calculated as the concentration of dissolved Ci required for half-maximal O_2_ evolution rate (Ma et al. 2011).

### Electrochromic shift measurements

ECS was recorded using the DUAL-PAM 100 system (Walz) with a P515/535 emitter/detector module as the absorbance difference signal 550 to 515 nm according to published protocols (Schreiber and Klughammer 2008). First, cells were dark adapted for 15 min, and then exposed to actinic red light of 660 *μ*mol photons m^−2^ s^−1^ for 10 min. Where indicated, cells were pretreated with DCCD as described (Joliot and Joliot 2001). The light was turned off and the ECS decay kinetics were recorded to determine total ECS (ECSt) representing total PMF size. Before each measurement, 3 pulses of 5-ms and 200,000 *μ*mol photons m^−2^ s^−1^ were applied and the signals were averaged to determine ECS_ST_, which was used to normalize the ECSt values of each measurement. Normalization was done by multiplying ECSt with a correction factor calculated as (maxECS_ST_/ECS_ST_), where maxECS_ST_ is the highest ECS_ST_ from all the measurements of a dataset, and ECS_ST_ is the ECS_ST_ corresponding to the measurement of the ECSt that is being normalized. To determine H^+^ conductivity of the thylakoid membrane through ATP synthase (g_H_^+^), the light was turned off at specific time points to record decay of the ECS signal during 620 ms dark intervals. The g_H_^+^ parameter was calculated as 1/τ (time constant for decay during the first 100 ms (Cruz et al. 2005). The total proton flux across the membrane was calculated as ν_H_^+^ = PMF × g_H_^+^ (Cruz et al. 2001).

### Transmission electron microscopy

After 4 days of growth in standard TP medium at 23 °C under 100 *μ*mol photons m^−2^ s^−1^, the cells were resuspended in TP medium (pH 7.4) for 2 h to adapt to the slightly higher pH before fixation. Cells were then resuspended in 2.5% (w/v) glutaraldehyde in TP (pH 7.4) and incubated for 1 h at 25 °C with gentle shaking followed incubation at 4 °C for ∼24 h. Fixed samples were briefly rinsed in distilled water for 15 min. After osmification (2%) for 2 h, samples were rinsed again in distilled water for additional 10 min, then dehydrated using a graded series of ethanol (Molar Chemicals Kft) concentrations (50%, 70%, 90%, 96% and 100%, v/v) for 10 min. Afterwards, samples were proceeded through propylene-oxide (Molar Chemicals Kft), then embedded in an epoxy-based resin mixed with propylene-oxide, Durcupan ACM (Sigma-Aldrich) (3:1. 1:1, 1:3; 3 × 1 h). Samples were then proceeded through the same epoxy-based resin but without propylene-oxide (2 × 1 h, 1 × overnight). After overnight polymerization at 56 °C (48 h), resin blocks were etched, and 80 nm thick ultrathin sections were cut using an Ultracut UCT ultramicrotome (Leica). Sections were mounted on a 100-mesh hole copper grid (Electron Microscopy Sciences), and contrast of the samples was enhanced by staining with 2% (w/v) uranyl acetate in 50% (v/v) ethanol (15 min, Molar Chemicals Kft, Electron Microscopy Sciences) and 2% (w/v) lead citrate in distilled water (10 min, Electron Microscopy Sciences). Ultrathin sections from the samples were screened at 3000 to 4,000× magnification on a JEM-1400 Flash transmission electron microscope (JEOL) until at least 10 cross-sections were identified from each sample. Images of WT, the *pht4-9* mutants and the complemented C1 strain were recorded at 8,000× magnification using a 2k × 2k Matataki (JEOL) scientific complementary metal-oxide-semiconductor camera.

### Statistical analyses

Presented data are means ± SEM of 3 to 4 biological replicates. Statistical analyses to compare the means among different genotypes and treatments were performed using Tukey one-way ANOVA. Statistically significant differences were considered at *P* < 0.05.

### Accession numbers

Sequence data in this article were obtained from Phytozome (Goodstein et al. 2012) version 13 under the following accession number: Na^+^-dependent inorganic phosphate cotransporter: Cre09.g396950.The accession numbers for other genes mentioned in this article are provided in Supplementary Fig. S1.

## Supplementary Material

kiaf158_Supplementary_Data

## Data Availability

All data are incorporated into the article and its online Supplementary material.

## Supplementary data

The following materials are available in the online version of this article.

**Supplementary Figure S1.** Amino acid sequence alignment of PHT4 members in *Chlamydomonas reinhardtii* (CrPHT4) and *Arabidopsis thaliana* (AtPHT4).

**Supplementary Figure S2.** Transmission electron microscopic images of wild type, the *pht4-9* mutants and the complemented C1 strain grown under photoautotrophic conditions.

**Supplementary Figure S3.** RT-qPCR for cell division marker genes.

**Supplementary Figure S4.** Chlorophyll *a* fluorescence transients for determination of the qE component of NPQ shown in Fig. 5E.

**Supplementary Figure S5.** Chlorophyll *a* fluorescence measurements for determination of the qT component of NPQ shown in Fig. 5F.

**Supplementary Figure S6.** The proton motive force and ATP synthase activity during illumination under CO_2_-limiting conditions.

**Supplementary Figure S7.** Effect of DCCD on the PMF and the ATP synthase activity under CO_2_-limiting conditions.

**Supplementary Figure S8.** CrPHT4-9 is not required for photochemistry and photoprotection under Pi- and CO_2_-limiting conditions.

**Supplementary Figure S9.** The proton motive force, ATP synthase activity and ATP content under Pi- and CO_2_-limiting conditions.

**Supplementary Figure S10.** Photosynthetic oxygen evolution versus Ci curves for determination of the *K*_1/2_(*C_i_*) shown in Figs. 7, D and 8D.

**Supplementary Figure S11.** The effect of air levels of CO_2_ and high (2%) CO_2_ on the cell number after 4 days in standard TP medium in a PSI Multi-Cultivator photobioreactor.

**Supplementary Table S1.** Growth and photosynthetic parameters under Pi-replete and Pi-limiting conditions.

**Supplementary Table S2.** Primers used in this work.

**Supplementary Table S3.** Electroporation settings for NEPA21.

## Dive Curated Terms

The following phenotypic, genotypic, and functional terms are of significance to the work described in this paper:

PHT4;1 Gramene: At2g29650
PHT4;1 Araport: At2g29650

PHT4;4 Gramene: At4g00370
PHT4;4 Araport: At4g00370

PHT4;5 Gramene: At5g20380
PHT4;5 Araport: At5g20380
