# Supplementary material for: The thylakoid- and pyrenoid-localized phosphate transporter PHT4-9 is essential for photosynthesis in Chlamydomonas
Source: Plant Physiol. 2025 Apr 24;198(1):kiaf158. doi: 10.1093/plphys/kiaf158 (PMC12056506; doi:10.1093/plphys/kiaf158)
Supplement: kiaf158_Supplementary_Data [file kiaf158_supplementary_data.pdf]

```
AtPHT4;5        ---------------------------------------MARLTLRPHNHFFSSPIYAHK
CrPHT4-9        MVQPVSVLTPGVQSGLGGLGASRPGASQAWPRRPAVLSTFLGTSMLWRGHRAASSTGGHV
CrPHT4-7        MLKLKAGPGVAQQRAPSGCAHVPCLRPLSAPAAGVQARSLSWAGVSGSPCRWLAPRHHRL
AtPHT4;1        -------------MNARALLCSSNIHSLYTSNRPPEKTSSS-----------RSLRNLKP
AtPHT4;4        -------------MALGGLISNRNFGSFIGSGNGCQRLGKSGAEVSKLFPNALLCRNHQP
                                                                          :

AtPHT4;5        QPFLSVYTIFPHHHQNPLIKSRVKCSASG--------------------TERVRESKKLP
CrPHT4-9        AQHVCSASSKSGRTVKITRRRGSGHSSSGGTGLVAVPADGPATSSGRYSGSMLGSASRTT
CrPHT4-7        QRRIAAASGGQQQPSDVRVNDGGSDAVSNG-------------------ASQNGTGSSAN
AtPHT4;1        SPKSLRVWIYPRNRSSVFRVLVRSSDKSES------------------SNSYYVEGDKVS
AtPHT4;4        LQASLHHESGHMRRSFGCFLQPRMDSVIRF------------------RNSIKINRSRAY
                            .                                     .     .

AtPHT4;5        PKDPIEDPKPQLPIPEVLSTETGFEQN-----WPPWKNIPQRYKLIGATSLAFVICNMDK
CrPHT4-9        SGRSTTSAPPQVREAEYLSSDNGSASDTNAFAWGWWESLPSRYKIIVGTAVSFVICNLDK
CrPHT4-7        GYQLSTNGAAAALPLPVASPEVAEVMN----------TRPHRWNVVVMIAVAFVLCNMDK
AtPHT4;1        GNND------------------VVSDSPSSIVLPWWEEFPKRWVIVLLCFSAFLLCNMDR
AtPHT4;4        YKSEESDITEGVVPSADGSAEAILVEGNLQNASPWWQQFPRRWVIVLLCFSSFLLCNMDR
                                          .            * *: ::     :*::**:*:

AtPHT4;5        VNLSIAIIPMSHQFGWSSSVAGLVQSSFFWGYALSQLPGGWLSKIFGGRKVLEIGVFTWS
CrPHT4-9        VNLSVCIIPMARDFGWSPTVAGLVQSAFFLGYMVCQLPGGYLNSRLGGRRILPAGVLLYS
CrPHT4-7        VNMSVAVIPMAAELGWSATERGLVSSSFFWGYSATQLPAGYISTKIGGAKVLAAGVALWS
AtPHT4,1        VNMSIAILPMSAEYGWNPATVGLIQSSFFWGYLLTQIAGGIWADTVGGKRVLGFGVIWWS
AtPHT4;4        VNMSIAILPMSQEYNWSSATVGLIQSSFFWGYLLTQILGGIWADKFGGKVVLGFGVVWWS
                **:*:.::**: : .*..:  **:.*:** **   *: .*     .**  :*  **  :*
AtPHT4;5        FATALVP-LLAGFMPGLIFSRILVGIGEGVSPSAATDLIARTIPVKERSRAVGFVFGGLS
CrPHT4-9        AATGVVP-WVAASVPALCVSRAFVGFGQATAPSAATDMIARAVPPSERARAVTFVFSGFH
CrPHT4-7        FGTLIAPPAAQTSLLALCATRVLVGLGEGFAPSAATAVLAKLVPSTERSRAVAAVWGGLD
AtPHT4;1        IATILTPVAAKLGLPYLLVVRAFMGVGEGVAMPAMNNILSKWVPVQERSRSLALVYSGMY
AtPHT4;4        FATIMTPIAARLGLPFLLVVRAFMGIGEGVAMPAMNNMLSKWIPVSERSRSLALVYSGMY
                 .* :.*      :  *   * ::*.*:. : .* . :::: :*  **:*::  *:.*:
AtPHT4;5        LGSVMGLLLAPPIIETFNWESVFYLFGLLGVGWFVGFQFLNEEEVSYKGNEISTSHKSEN
CrPHT4-9        VGSILGLLTAPWLIQHYGWQSVFVTFGALGFVWWLWFEQGIMSRIKAEEPDFATRLVSDS
CrPHT4,7        VGSAVGLLLCGPLIRMFGWPSVFYLFAVLGLVWVAAWPLVQPDKMDPDMIAENKKKEQER
AtPHT4;1        LGSVTGLAFSPFLIHQFGWPSVFYSFGSLGTVWLTLWLTKAESSPLEDPTLLPEER----
AtPHT4;4        LGSVTGLAFSPMLITKFGWPSVFYSFGSLGSIWFLLWLKFAYSSPKDDPDLSEEEK----
                :**  **  .  :*  :.* ***  *. **  *   :     .    .
AtPHT4;5        -------------------------------------ATKEELGSSLKEIPWKSFFQSPA
CrPHT4-9        RRIAPMTDANSVTSASSSVVVTNKAVANRGAGAAAAAAAAAAAAAEPPPMPWRAFLRSTP
CrPHT4-7        ATRLALAQANEVAASALESVDETP--------VSATYAKLEKSLPPDGKVPWGEFFRSPP
AtPHT4;1        ----------------------------------KLIADNCASKEPVKSIPWRLILSKPP
AtPHT4;4        ----------------------------------KVILGGSKPREPVTVIPWKLILSKPP
                                                 :**  :: ...

AtPHT4;5        VWAMIYTHFCGSWGHYTCLSWLPTYFSEALSLNLTEAAWVSILPPLASIVVTSLASQFAD
CrPHT4-9        VWALAYTHFANNWFHYTMLAWLPTYFVDTLSVDLLHASQTALLPPLAGIAASAAAGAAAD
CrPHT4-7        VWAVTVAHFCFNWGYYTLLAWLPSYFELALGLNVERSSFLTLIPYIAMTAMMPLVGPVAD
AtPHT4;1        VWALISCHFCHNWGTFILLTWMPTYYHQVLKFNLMESGLLSVFPWMTMAISANAGGWIAD
AtPHT4;4        VWALIISHFCHNWGTFILLTWMPTYYNQVLKFNLTESGLLCVLPWLTMAVFANIGGWIAD
                ***:   **. .*  :  *:*:*:*:  .* .:: .:.   ::* ::        .  **
AtPHT4;5        YLITNGVDTTTVRKICQTIAFVAP-AICMTLSSVDIGLPP-----------------WEI
CrPHT4-9        ALISRGVRVPVVRKLVQGVAFLVPTALLLAACTPAIADDS-----------------GLT
CrPHT4-7        GWVKNGVPLTRVRKICQGIAFVGPAVCMIACAILTPAAAATAKAATTGVSAAGPVLTAVL
AtPHT4;1        TLVSRGFSVTNVRKIMQTIGFLGPAFFLTQLKHIDSPTMA--------------------
AtPHT4;4        TLVSRGLSITNVRKIMQSIGFLGPAFFLSQLSHVKTPAMA--------------------
                  :..*.  . ***: * :.*: *               .
AtPHT4;5        VGILTAGLALSSFALSGLYCTHQDISPEYASILLGITNTVGAVPGIVGVALTGFLLDSTH
CrPHT4-9        VAAITVALGLSSFSLAGLFCTHQDMSPKYAPILLGLTNTTAAVPGLLGVASVGYLFEQTQ
CrPHT4-7        VGLMSVAFALGAWSRAGLYCNHQDLSPKYASALLGITNTAGAIPGVLGVTMAGYLLDTTA
AtPHT4;1        VLCMACSQGTDAFSQSGLYSNHQDIAPRYSGVLLGLSNTAGVLAGVLGTAATGHILQHGS
AtPHT4;4        VLCMACSQGSDAFSQSGLYSNHQDIGPRYAGVLLGLSNTAGVLAGVFGTAATGYILQRGS
                *  :: . . .::: :**:..***:.*.*:  ***::**...:.*:.*.: .*.:::
AtPHT4;5        SWTMSLFVPSIFFYLTGTVVWLAFASSEPQTFRKEDS-----------------------
CrPHT4-9        SWEAALFLPSAFFMVTAAAVYTLWGRNDPIDFDAADNGPFRFESRIAAIKKAVDDAFDFG
CrPHT4-7        SWANALFIPTAICQLFGAAVYTWLASSERQSWS---------------------------
AtPHT4;1        --WDDVFTISVGLYLVGTVIWNLFSTGEKIID----------------------------
AtPHT4;4        --WDDVFKVAVALYLIGTLVWNLFATGEKILD----------------------------
                     :*  :    : .: ::   . .:
AtPHT4;5        ------------------------------------------------------------
CrPHT4-9        QKPSAGTDVEREVDVPEVPPRPPSSLLGPLADLVRGTSSATASGDDGDEGGSVRRRVGGA
CrPHT4-7        ------------------------------------------------------------
AtPHT4;1        ------------------------------------------------------------
AtPHT4;4        ------------------------------------------------------------

AtPHT4;5        ------------------------------------------------------------
CrPHT4-9        ARNAAGSGRRTSGSSSSGSGRRTTVGSQSRRSTGGSQSKATGGGRRVQEAEEEGPGGVWE
CrPHT4-7        ------------------------------------------------------------
AtPHT4;1        ------------------------------------------------------------
AtPHT4;4        ------------------------------------------------------------

AtPHT4;5        ------------
CrPHT4-9        RLRALLGQPREY
CrPHT4-7        ------------
AtPHT4;1        ------------
AtPHT4;4        ------------
```

Supplementary Figure S1. Amino acid sequence alignment of PHT4 members in *Chlamydomonas reinhardtii* (CrPHT4) and *Arabidopsis thaliana* (AtPHT4). The accession numbers are: *Cre09.g396950* (CrPHT4-9), *Cre16.g663600* (CrPHT4-7), *At2g29650* (AtPHT4;1), *At4g00370* (AtPHT4;4), and *At5g20380* (AtPHT4;5). The alignment was performed using ClustalW 2.1. The identical (*), conserved (:) and semi-conserved (.) residues are indicated below the alignment. Key conserved residues are highlighted: Pi binding and translocation (blue), Na^+ binding (red), protein conformational changes (magenta). For CrPHT4-9; the predicted chloroplast transit peptide is coloured in orange and the bold orange residue is the amino acid after which the chloroplast transit peptide (according to TargetP) is cleaved. Transmembrane domains according to DeepTHMMM are indicated on a grey background and Rubisco-binding motif on a yellow background.

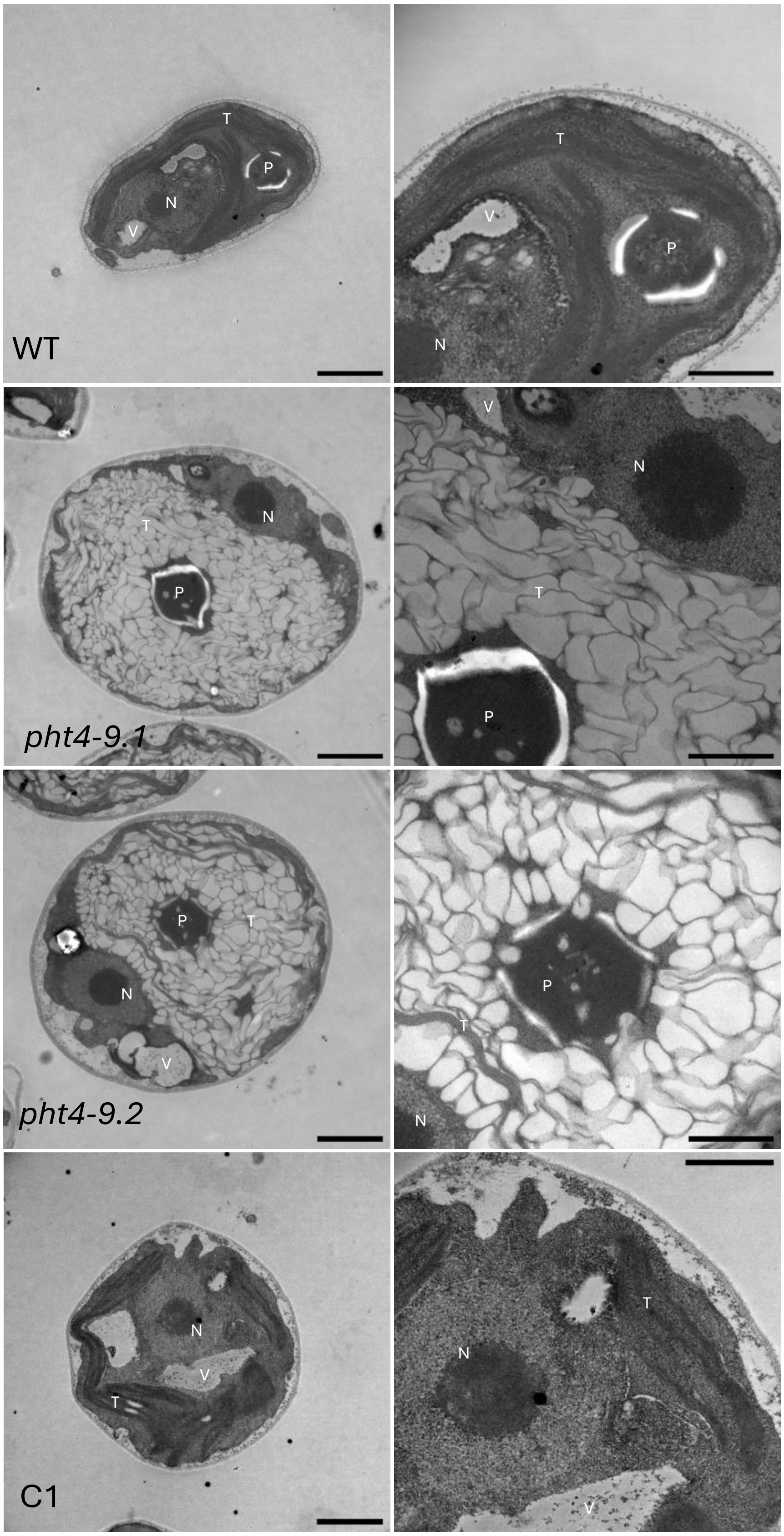

Supplementary Figure S2. Transmission electron microscopic images of wild type (WT), the *pht4-9* mutants and the complemented C1 strain grown under photoautotrophic conditions. Chlamydomonas cultures of wild type (WT), two *pht4-9* mutants and the complemented C1 strain were grown for four days in standard TP in the light at 100 µmol photons m^{-2} s^{-1} and air levels of CO_2. Samples were fixed and analyzed by transmission electron microscopy as described in Materials and Methods. Representative images for at least ten inspected sections for each sample are shown. *Left:* overall cell structure with key features labelled such as the nucleus (N), pyrenoid (P), the thylakoid membranes (T), and the vacuole (V), scale bar 2 µm. *Right*: the same cells at a higher magnification, scale bar 1 µm. The *pht4-9* mutans displayed enlarged cells and swollen thylakoids occupying a substantial portion of the chloroplast, while the complemented C1 strain resembled WT.

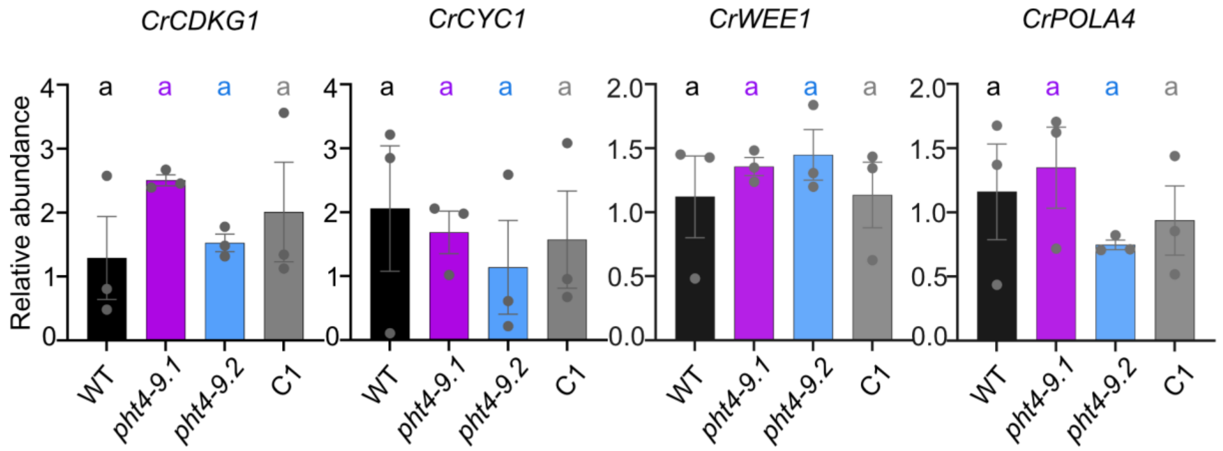

Supplementary Figure S3. RT-qPCR for cell division marker genes. RNA was extracted from Chlamydomonas cultures of wild type (WT), two *pht4-9* mutants and the complemented C1 strain grown for four days in standard TP under light at 100 µmol photons m^{-2} s^{-1} and air levels of CO_2. Transcript levels of four marker genes for cell division were determined by RT-qPCR relative to one control gene (Supplementary Table S2). The data are means ± SEM of three biological replicates. Identical letters indicate statistically non-significant differences among the genotypes with $P > 0.05$ using Tukey one-way ANOVA. *CDKG1*, cyclin-dependent kinase 1; *CYC1*, cyclin C1; *WEE1*, G2 checkpoint kinase; *POLA4*, DNA polymerase alpha 4.

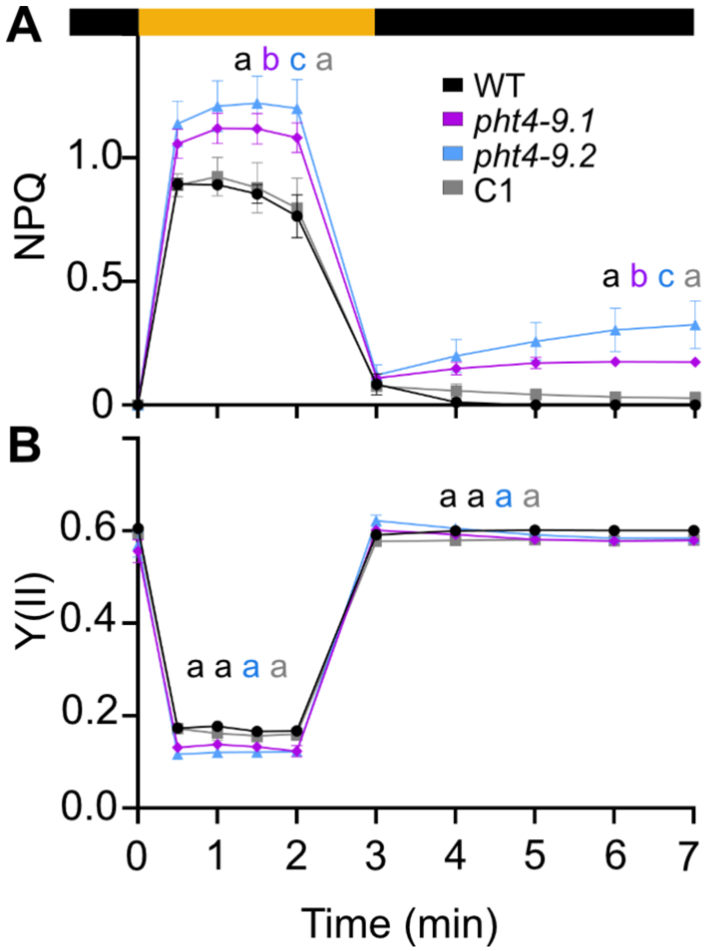

Supplementary Figure S4. Chlorophyll *a* fluorescence transient for determination of the qE component of NPQ shown in Fig. 5E. Chlamydomonas cultures of wild type (WT), two *pht4-9* mutants and the complemented C1 strain were grown for four days in standard TP in the light at 100 µmol photons m^{-2} s^{-1} and air levels of CO_2. **A**) The non-photochemical quenching (NPQ) in dark-adapted samples exposed to 325 µmol photons m^{-2} s^{-1} illumination for 2 min followed by 5 min in darkness. **B**) Photosystem II efficiency (Y(II) obtained in identical conditions as in **A**). The qE values plotted in Fig. 5E were calculated as described in Materials and Methods. The data are means ± SEM of three biological replicates. Different letters indicate statistically significant differences among the genotypes with $P < 0.05$ using Tukey one-way ANOVA.

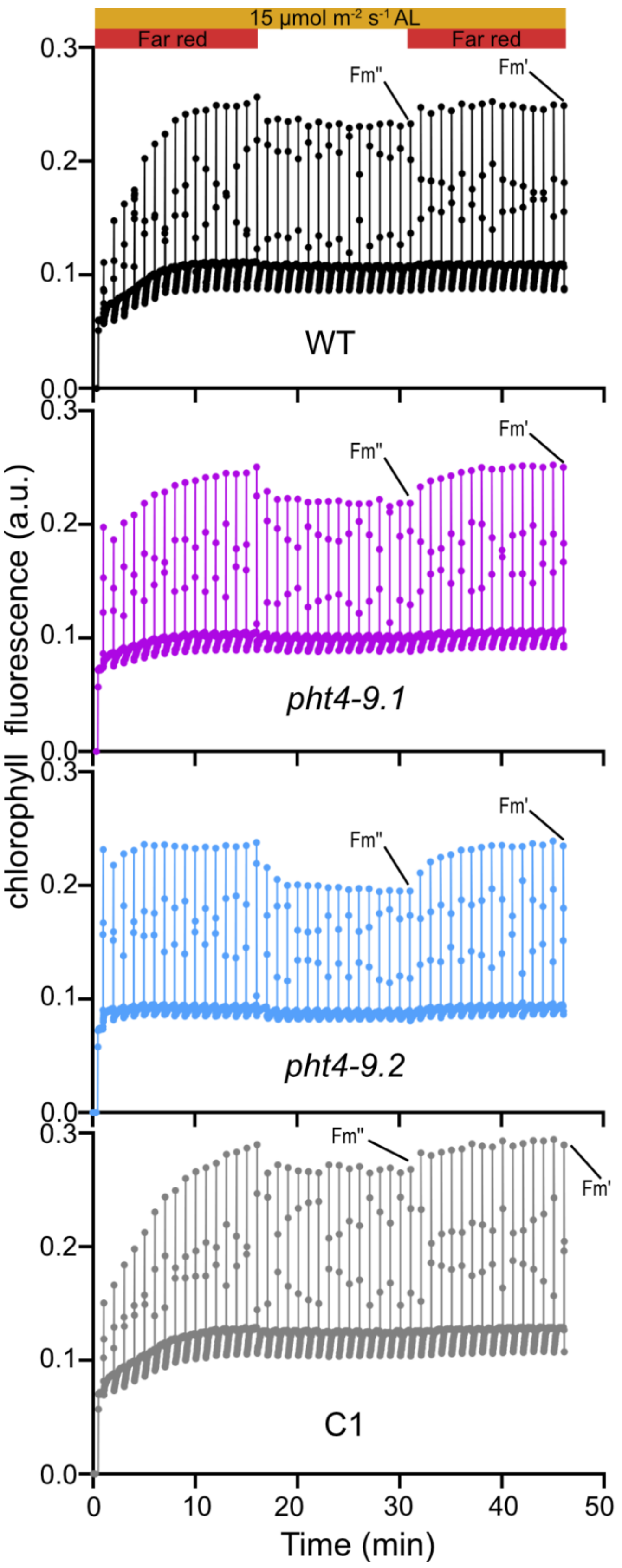

Supplementary Figure S5. **Chlorophyll *a* fluorescence measurements for determination of the qT component of NPQ shown in Fig. 5F.** Chlamydomonas cultures of wild type (WT), *pht4-9* mutants and the complemented C1 strain were grown for four days in standard TP in the light at 100 µmol photons m^{-2} s^{-1} and air levels of CO_2. Dark-adapted cultures were illuminated with actinic red light (AL, 15 µmol photons m^{-2} s^{-1}) and far red (FR) light (255 µmol photons m^{-2} s^{-1}) for 15 min (phase 1). Then, the FR light was turned off and only red-light illumination was employed for 15 min to induce state II (phase 2). Finally, the red light – far-red light combination was used again for 15 min to drive the state II - state I transition (phase 3). The curves for each of the genotype are presented in separate plots. The values for the qT parameter plotted in Fig. 5F were calculated as described in Materials and Methods.

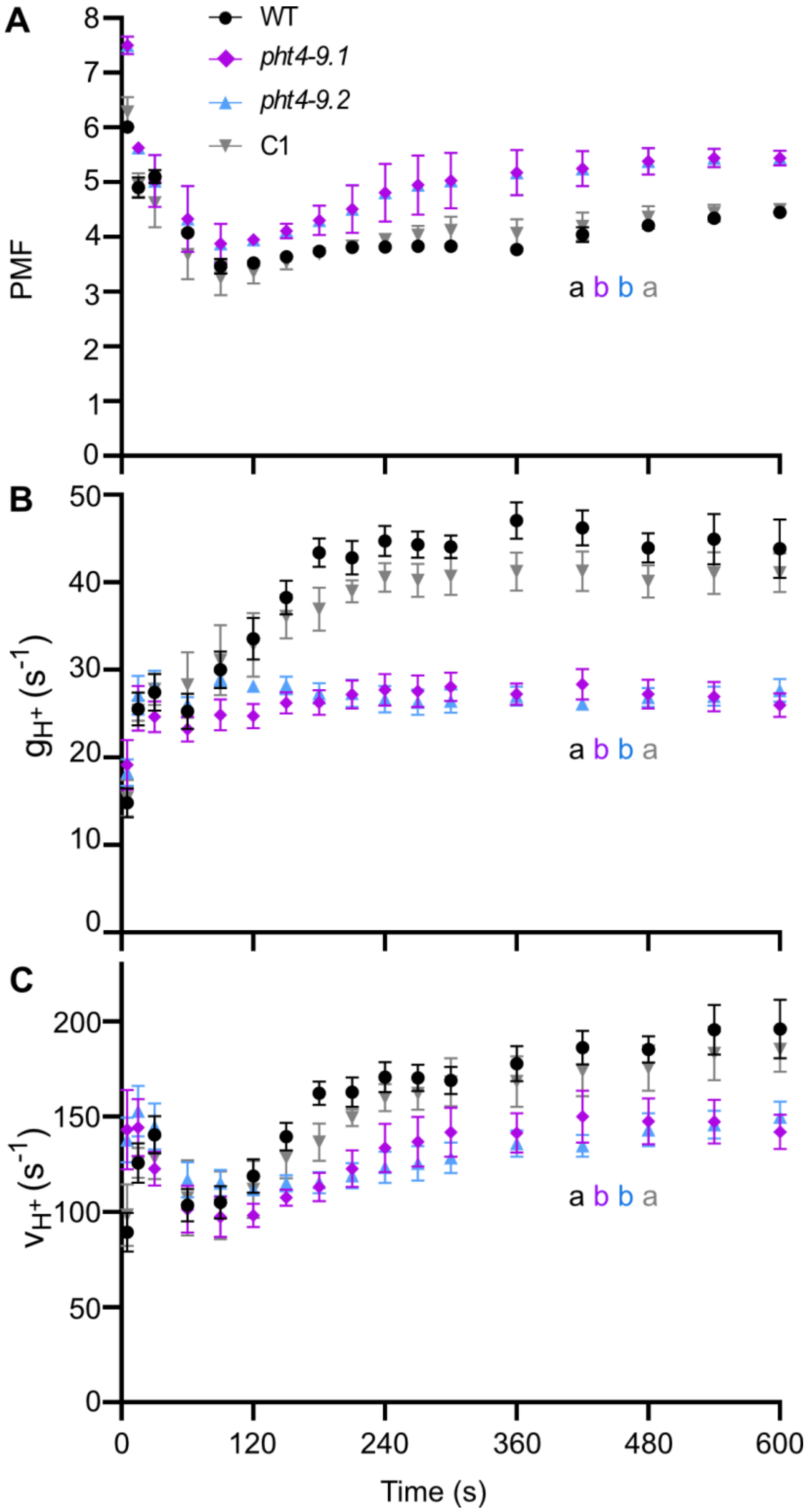

Supplementary Figure S6. The proton motive force and ATP synthase activity during illumination under CO_2-limiting conditions. Chlamydomonas cultures of wild type (WT), two *pht4-9* mutants and the complemented C1 strain were grown for four days in standard TP medium in the light at 100 µmol photons m^{-2} s^{-1} and air levels of CO_2, dark-adapted for 15 min, illuminated for 10 min at 660 µmol photons m^{-2} s^{-1}, followed by electrochromic shift decay measurements every 30 s. **A**) Proton motive force (PMF). **B**) H^+ conductivity of the ATP synthase (g_{H^+}). **C**) H^+ flux through the ATP synthase (v_{H^+}). The data are means ± SEM of three biological replicates. Different letters indicate statistically significant differences among the genotypes with $P < 0.05$ using Tukey one-way ANOVA.

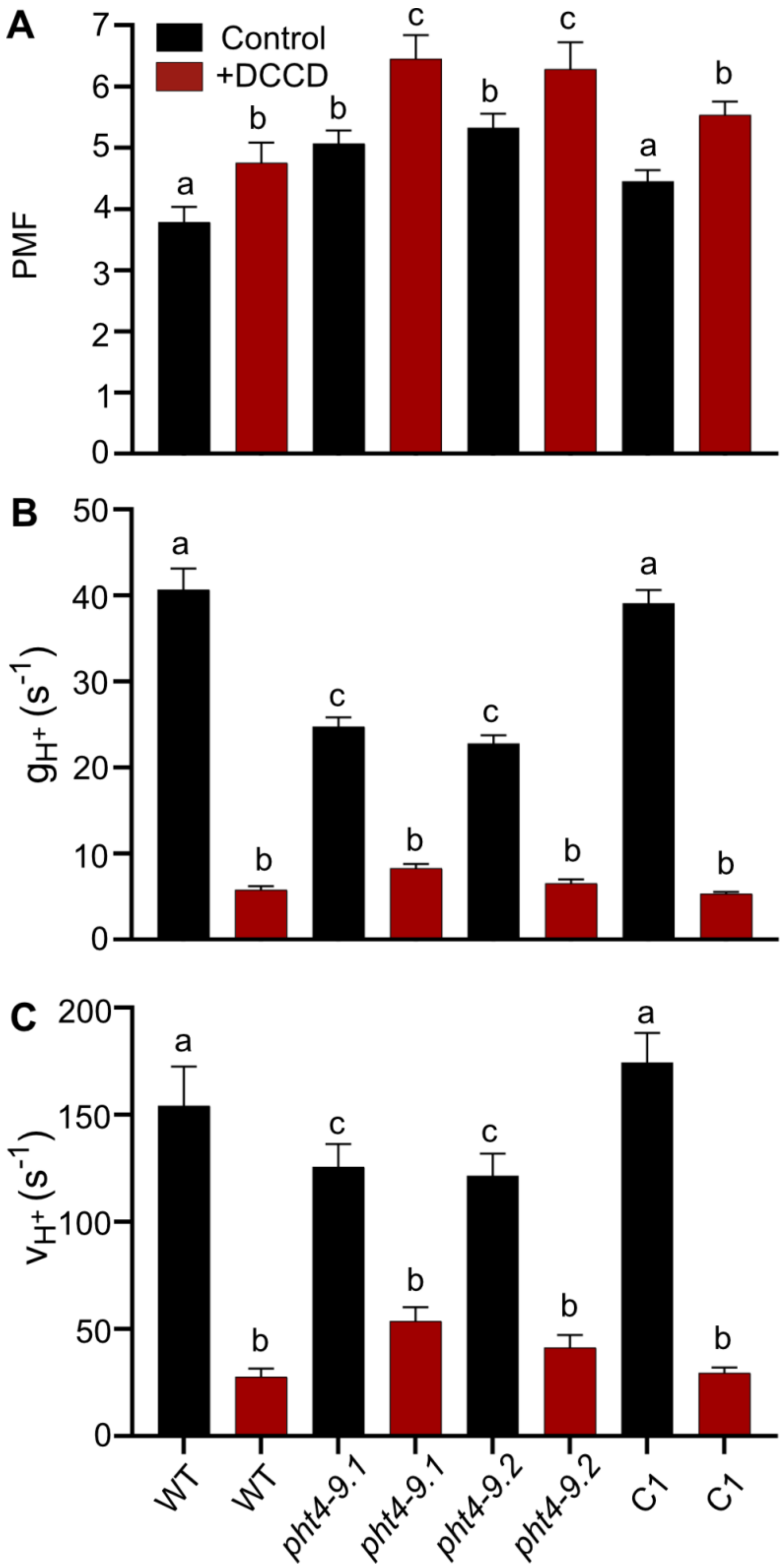

Supplementary Figure S7. Effect of DCCD on the proton motive force and the ATP synthase activity under CO_2-limiting conditions. Chlamydomonas cultures of wild type (WT), two *pht4-9* mutants and the complemented C1 strain were grown for four days in standard TP medium in the light at 100 µmol photons m^{-2} s^{-1} and air levels of CO_2, dark-adapted for 15 min in the presence of 18 µM DCCD, illuminated for 10 min at 660 µmol photons m^{-2} s^{-1}, followed by electrochromic shift decay measurements. The parameters were obtained at the end of the 10 min illumination and calculated as described in Materials and Methods. **A**) Proton motive force (PMF). **B**) H^+ conductivity of the ATP synthase (g_H^+). **C**) H^+ flux through the ATP synthase (v_H^+). DCCD reduced PMF, g_H^+ and v_H^+ to a residual level and the difference observed among the untreated cultures became statistically nonsignificant. The data are means ± SEM of three biological replicates. Different letters on top of bars indicate statistically significant differences among the genotypes with $P < 0.05$ using Tukey one-way ANOVA.

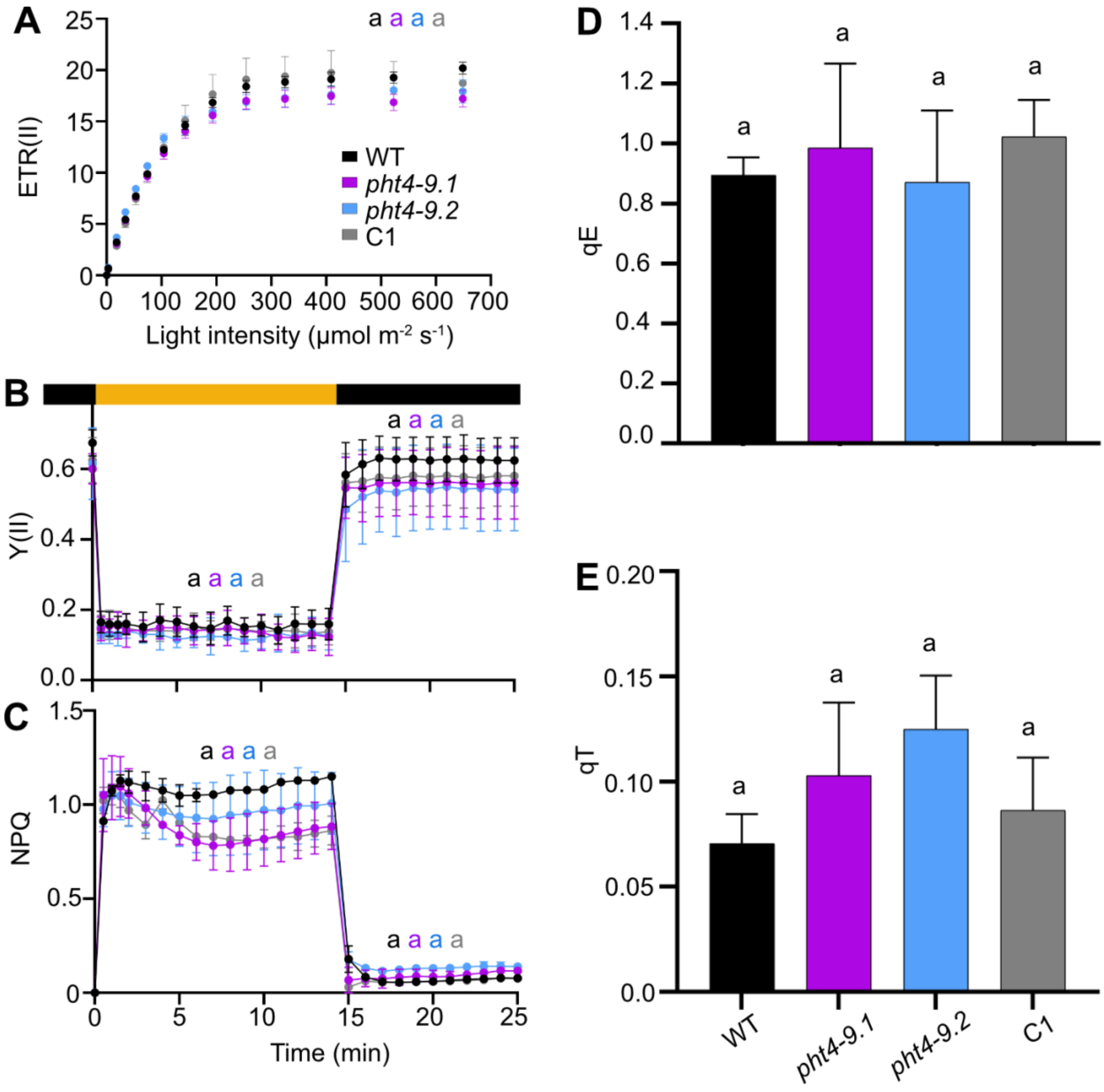

Supplementary Figure S8. CrPHT4-9 is not required for photosynthesis and photoprotection under Pi- and CO_2-limiting conditions. Chlamydomonas cultures of wild type (WT), two *pht4-9* mutants and the complemented C1 strain were grown for four days in TP medium with 2% Pi (corresponding to 20.4 µM final concentration of Pi) in the light at 100 µmol photons m^{-2} s^{-1} and air levels of CO_2. **A**) Light response curve of electron transport rate of photosystem II (ETR(II)) versus irradiation. **B**) Photosystem II efficiency (Y(II) in dark-adapted samples exposed to 325 µmol photons m^{-2} s^{-1} illumination for 15 min followed by 10 min in darkness. **C**) Non-photochemical quenching (NPQ) obtained under identical conditions as in **B**). **D**) The energy-dependent fast NPQ component (qE) as determined after 2 min of illumination at 325 µmol photons m^{-2} s^{-1}. **E**) The state transition-dependent NPQ component (qT) determined upon transition from red- to far-red light. The data are means ± SEM of three biological replicates. Identical letters indicate statistically non-significant differences among the genotypes with $P > 0.05$ using Tukey one-way ANOVA.

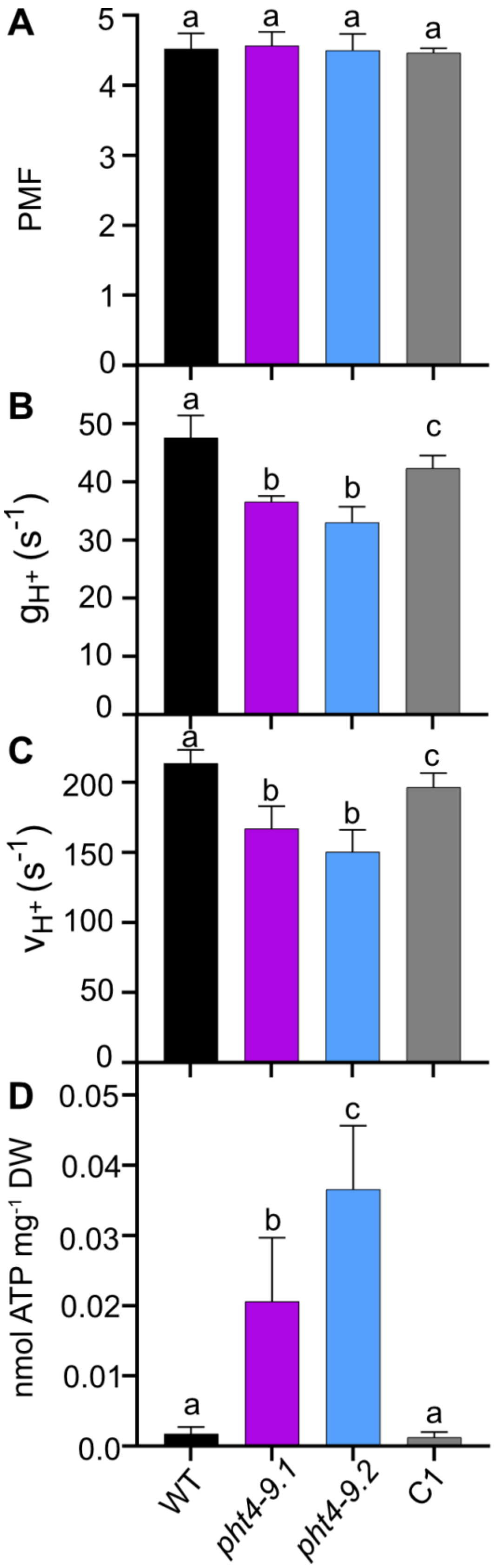

Supplementary Figure S9. **The proton motive force, ATP synthase activity and ATP content under Pi- and CO_2-limiting conditions.** Chlamydomonas cultures of wild type (WT), two *pht4-9* mutants and the complemented C1 strain were grown for four days in TP medium with 2% Pi (corresponding to 20.4 µM final concentration of Pi) at 100 µmol photons m^{-2} s^{-1} and air levels of CO_2, dark-adapted for 15 min, illuminated for 10 min at 660 µmol photons m^{-2} s^{-1}, followed by electrochromic shift decay measurements. **A**) proton motive force (PMF). **B**) H^+ conductivity of the ATP synthase ($g_H{}^+$). **C**) H^+ flux through the ATP synthase ($v_H{}^+$) at the end of the illumination. **D**) Total ATP content was determined as described in Materials and Methods and expressed per dry weight (DW). The data are means ± SEM of three biological replicates. Different letters above the bars indicate statistically significant differences among the genotypes with $P < 0.05$ using Tukey one-way ANOVA.

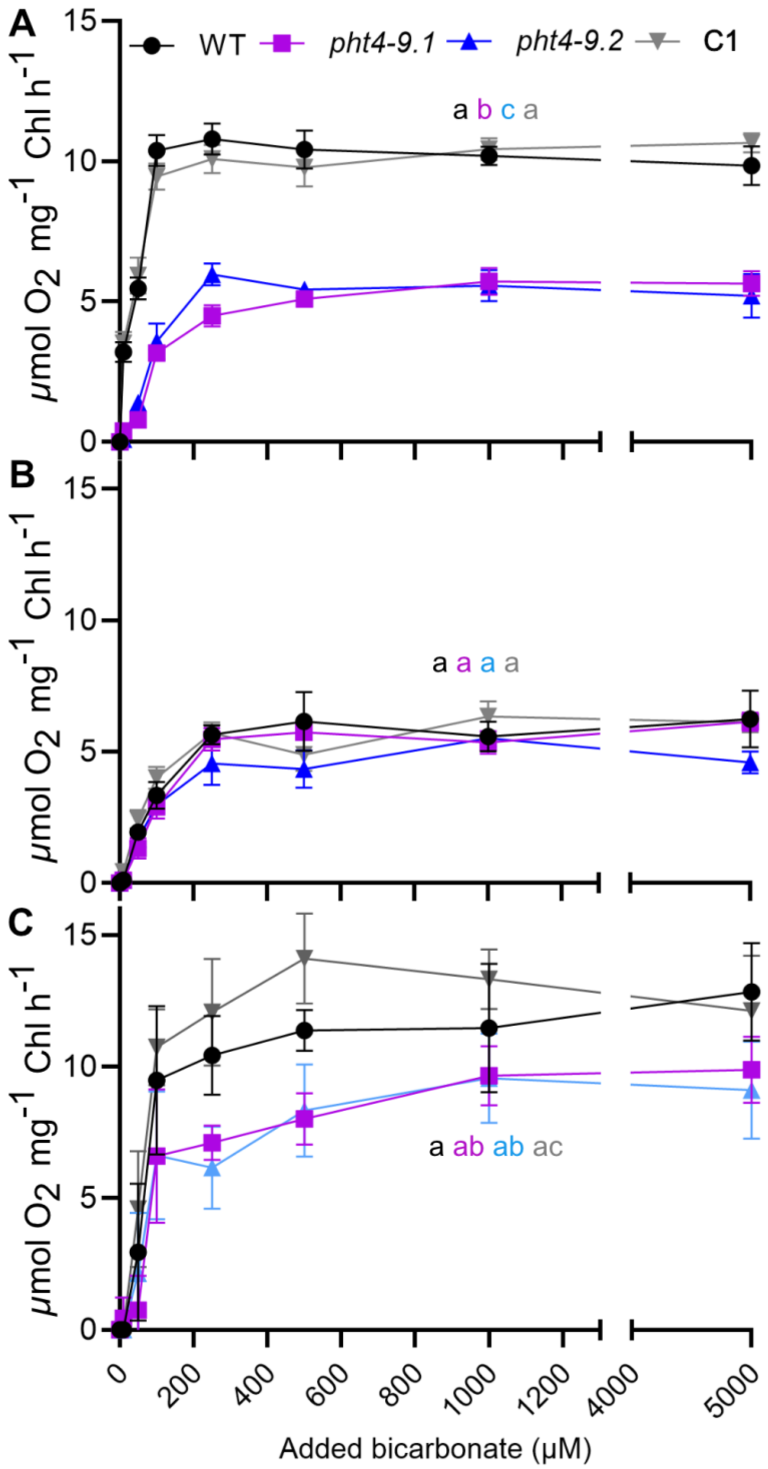

Supplementary Figure S10. **Photosynthetic oxygen evolution versus Ci curves for determination of the $K_{1/2}(C_i)$ shown in Figs. 7D and 8D.** Chlamydomonas cultures of wild type (WT), two *pht4-9* mutants and the complemented C1 strain were grown for four days in the light at 100 µmol photons m^{-2} s^{-1} in TP medium at the following Pi and CO_2 concentrations: **A**) standard TP and air levels of CO_2; **B**) TP with 2% Pi (corresponding to 20.4 µM final concentration of Pi) and air levels of CO_2, and **C**) standard TP and air enriched with 2% CO_2. Oxygen evolution was measured at pH 7.4 and a light intensity of 300 µmol photons m^{-2} s^{-1} in the presence of increasing $NaHCO_3$ concentrations. The curves were used to calculate the $K_{1/2}(C_i)$ values plotted in Figs. 7D and 8D. The data are means ± SEM of three biological replicates. Different letters indicate statistically significant differences among the genotypes with $P < 0.05$ using Tukey one-way ANOVA. We note that the O_2 evolution rates for the cell wall deficient wild-type CC-4533 (WT) strain in our study are lower than those reported for walled wild-type strains such as D66 (Ma et al., 2011, Mukherjee et al. 2019). Nevertheless, the calculated $K_{1/2}(C_i)$ values for CC-4533 are in agreement with those previously reported for D66.

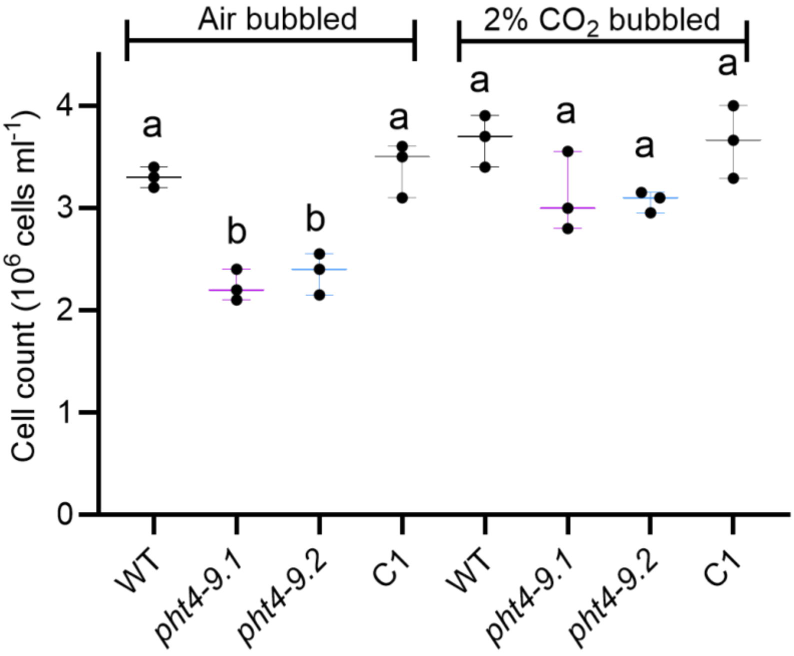

Supplementary Figure S11. **The effect of air levels of CO_2 and high (2%) CO_2 on the cell number after four days of growth in standard TP medium in a PSI Multi-Cultivator photobioreactor.** The plot for wild type (WT), two *pht4-9* mutants and the complemented C1 strain shows the minimum, median and maximum values (as lines) together with the data for three biological replicates (as filled circles). Different letters indicate statistically significant differences among the genotypes with $P < 0.05$ using Tukey one-way ANOVA.

Supplementary Table S1. Growth and photosynthetic parameters under Pi-replete and Pi-limiting conditions.

	1 mM Pi				20.4 µM Pi			
**Parameter**	**WT**	***pht4-9.1***	***pht4-9.2***	**C1**	**WT**	***pht4-9.1***	***pht4-9.2***	**C1**
Growth rate (d^{-1})	0.373 ± 0.002[a]	0.344 ± 0.002[b]	0.299 ± 0.008[c]	0.375 ± 0.000[a]	0.337 ± 0.001[b]	0.306 ± 0.002[c]	0.261 ± 0.005[c]	0.328 ± 0.004[b]
F_v/F_m	0.612 ± 0.004[a]	0.540 ± 0.004[b]	0.558 ± 0.003[b]	0.607 ± 0.001[a]	0.611 ± 0.004[a]	0.594 ± 0.006[a]	0.603 ± 0.003[a]	0.618 ± 0.007[a]
ETR (II)	14.27 ± 0.34[a]	11.24 ± 0.44[c]	11.70 ± 0.26[c]	13.75 ± 0.18[a]	12.23 ± 0.29[b]	11.88 ± 0.47[c]	13.36 ± 0.36[d]	12.48 ± 0.54[b]
qE	0.823 ± 0.044[a]	1.043 ± 0.044[b]	1.107 ± 0.080[b]	0.849 ± 0.035[a]	0.894 ± 0.030[a]	0.985 ± 0.141[ab]	0.871 ± 0.120[ab]	1.023 ± 0.061[a]
qT	0.077 ± 0.001[a]	0.127 ± 0.008[b]	0.169 ± 0.010[b]	0.070 ± 0.007[a]	0.070 ± 0.006[a]	0.103 ± 0.016[ab]	0.125 ± 0.012[ab]	0.086 ± 0.011[ab]
PMF	4.46 ± 0.07[a]	5.44 ± 0.11[b]	5.44 ± 0.11[b]	4.50 ± 0.08[a]	4.52 ± 0.22[a]	4.57 ± 0.20[a]	4.56 ± 0.20[a]	4.42 ± 0.13[a]
g_H^+ (s^{-1})	43.87 ± 3.04[a]	25.98 ± 1.21[b]	27.65 ± 1.21[b]	41.09 ± 2.02[a]	47.55 ± 1.92[a]	36.58 ± 0.59[c]	33.03 ± 1.56[c]	42.31 ± 1.27[a]
ATP (nmol mg^{-1} DW)	0.037 ± 0.003[a]	0.035 ± 0.001[a]	0.050 ± 0.001[a]	0.040 ± 0.005[a]	0.001 ± 0.000[b]	0.020 ± 0.004[a]	0.036 ± 0.004[a]	0.001 ± 0.000[b]

Chlamydomonas cultures of wild type (WT), two *pht4-9* mutants and the complemented C1 strain were grown in standard TP (1 mM Pi) or TP with 2% Pi (corresponding to 20.4 µM final concentration of Pi) in the light at 100 µmol photons m^{-2} s^{-1} and air levels of CO_2. Presented data were extracted from Figs. 4–7 and Supplemental Figs. S8 and S9. The growth rates were calculated using cell counts values at day 10. The electron transport rates of photosystem II (ETR(II)) correspond to the light intensity used during growth. F_v/F_m, maximum PSII quantum yield; qE and qT, pH-dependent and state transition-dependent components of non-photochemical quenching; PMF, proton motive force; g_H^+, H^+ conductivity of the ATP synthase; DW, dry weight. The data are means ± SEM of three biological replicates. Different letters indicate statistically significant differences among the genotypes and treatments with $P < 0.05$ using Tukey one-way ANOVA.

Supplementary Table S2. **Primers used in this work.**

Gene name	Application	Forward sequence	Reverse sequence
CrPHT4-9	PCR, *pht4-9.1*	AGTCAGGTTCAGGGTTCAGA	AATGCGTTGGTGTCACTAGC
	PCR, *pht4-9.2*	CCATCAGTCAGGTTCAGGGT	CGATGATTTTGTAGCGGGAT
	PCR, *CIB* cassette insertion	GCACCAATCATGTCAAGCCT	GACGTTACAGCACACCCTTG
	PCR, *pht4-9.1* complementation	CGTTGCACAGGTTTGAGTCG	ATGGTCCTTGTAGTCGCCAC
	RT-qPCR, *pht4-9.1* complementation	CGCTAGGCTTCGTGTGG	TGTTGGCGTCGGTCATG
	Recombineering	GGAGATCTGGGTGGCTCCG	GAAGATCCTTTGATCTTTTCTACGGG
	Homology arm	TCCCTTCAATTGTAATTCGCATCCTTAGTGTGGTACGGAATGCGATACAT	
CrCDKG1	RT-qPCR, cell division	TGGTACACAGCATTGAGAACG	CGGGAAGTTCTCCGAGTAGTA
CrCYC1	RT-qPCR, cell division	GGACTCGCTGAACCACGAAGA	CAGGATCAGGTTGCCCAGGTA
CrWEE1	RT-qPCR, cell division	CAAGCGGCGCGTGCAGTTCA	GAGGTGGTCGCCGCGAGCTG
CrPOLA4	RT-qPCR, cell division	CGAGTTCTGCTTTACGCTCCAAGG	CCCTTGTACATGGCGAACCAGGAA
CrGBLP	RT-qPCR control	ACTGGCTGTGATTGTGCTTCAGG	TGTCTGCTGCTGCACCTTTACG
Crβ-tubulin2	RT-qPCR control	ACTGGCTGTGATTGTGCTTCAGG	TGTCTGCTGCTGCACCTTTACG

Supplementary Table S3. Electroporation settings for NEPA21.

	Voltage (V)	Pulse length (ms)	Pulse interval (ms)	Number of pulses	Decay rate (%)	Polarity
Poring	300	8	50	2	40	+
Transfer	20	50	50	1	-	+/-
